# Superlattice Architectures for Advancing Photothermal Catalysis: Mechanisms and Applications

**DOI:** 10.1002/advs.75682

**Published:** 2026-05-14

**Authors:** Yuzhao Wu, Xiaoguang Duan, Haijun Chen, Hongqi Sun, Jinqiang Zhang, Shaobin Wang

**Affiliations:** ^1^ School of Chemical Engineering Adelaide University Adelaide South Australia Australia; ^2^ School of Mechanical and Power Engineering Nanjing Tech University Nanjing Jiangsu China; ^3^ School of Molecular Sciences The University of Western Australia Perth Western Australia Australia

**Keywords:** photoelectrochemical effect, photothermal catalysis, photothermal effect, solar fuels, superlattice nanostructure

## Abstract

Photothermal catalysis has emerged as a powerful strategy to complement photocatalysis by harnessing full‐spectrum sunlight for the production of solar fuels and chemical upgrading. Despite its promise, practical implementation remains limited by inefficient management of light‐to‐electron/heat conversion and unclear catalytic mechanisms. Superlattice materials, featuring periodic structural order across atomic to macroscopic scales, offer tuneable sized crystals, controllable electronic structures, and unique catalytic functionalities. These attributes enable enhanced light capture, energy conversion, and highly efficient catalytic kinetics for photothermal catalysis. This review provides an overview of superlattice architectures in photothermal catalysis, with a systematic review of the mechanism at both atomic and macroscopic scales. Emphasis is placed on the mechanism of superlattice engineering in regulating key photothermal processes, including photo‐electron–phonon coupling and multi‐energy‐carrier dynamics. Advanced microscopic and operando characterization techniques are highlighted to elucidate reaction pathways and disentangle photothermal and photoelectrochemical contributions. Representative photothermal reactions are discussed to demonstrate how superlattice nanostructures enhance reactivity, selectivity, and product upgrading. Finally, challenges and future opportunities are outlined. This work aims to advance photothermal catalysis by introducing ultra‐fast, directional photon–electron–phonon transport channels and tailoring highly ordered surfaces and interfaces to steer solar‐driven catalysis toward more efficient, selective, and value‐oriented chemical transformations.

## Graphical abstract

This paper provides a timely overview of superlattice architectures in photothermal catalysis, with a systematic review on mechanisms at both atomic and macroscopic scales in different processes, challenges, and future opportunities.

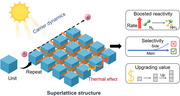

## 1 Introduction

Anthropogenic activities have profoundly perturbed global biogeochemical cycles, giving rise to escalating energy and environmental pressures. Massive consumption of fossil resources, together with the emissions of greenhouse gases (CO_2_, CH_4_, N_2_O) and reactive pollutants (NO_x_, volatile organic compounds, etc.), is accelerating climate change, regional air pollution, and ecosystem degradation [1, 2]. Modern society remains heavily dependent on high‐temperature thermocatalytic processes, such as Haber‐Bosch ammonia synthesis, fossil‐fuel reforming, and oxidative conversions, to supply fuels and commodity chemicals. While these technologies underpin global agriculture and industry, they are intrinsically energy‐intensive and carbon‐emissive, fundamentally conflicting with objectives for long‐term sustainability. Consequently, there is an urgent demand for transformative catalytic technologies capable of utilizing renewable energy to convert abundant small molecules (e.g., H_2_O, CO_2_, N_2_, O_2_, and simple organics) into value‐added fuels and chemicals in an efficient and environmentally benign manner.

Solar energy, as a clean and inexhaustible resource, has stimulated extensive research in photocatalysis over recent decades [3, 4, 5]. By harnessing solar photons, photocatalysis enables a wide range of redox reactions [6], including water splitting, CO_2_ reduction, N_2_ fixation, selective oxidation, and environmental remediation. Through rational engineering of active sites and photogenerated charge‐carrier dynamics, photocatalysts can selectively steer reaction pathways while suppressing undesired byproducts, offering a compelling platform for green chemical synthesis. Nevertheless, the intrinsically low efficiency of photocatalysis remains a critical limitation. Even for benchmark water splitting, the theoretical solar‐to‐hydrogen efficiency is capped at ∼24%, while state‐of‐the‐art experimental systems typically achieve < 10% [7]. For more complex small‐molecule conversions, solar‐to‐chemical efficiencies even fall below 1% [8, 9], severely impeding the large‐scale industrial deployment of purely photocatalytic processes.

Alternatively, photothermal catalysis, widely regarded as a next‐generation extension of photocatalysis, has therefore emerged as a promising frontier in solar energy conversion. Unlike conventional photocatalysis, photothermal catalysis enables direct utilization of full‐spectrum sunlight without auxiliary components or external energy inputs. Upon irradiation, photothermal catalysts can generate energetic hot carriers (EHCs) and localized hot spots, which synergistically promote catalytic reactions. The EHCs can overcome activation barriers under milder conditions and selectively drive reaction pathways toward desired products [10], while localized heating suppresses charge recombination [11], accelerates charge migration [12], and lowers reaction activation energies [13]. Moreover, photothermal effects may induce subtle modifications in the band structures of certain semiconductors, influencing charge kinetics and reaction thermodynamics. Despite these advantages, current catalyst design strategies remain rooted in conventional photocatalytic or thermocatalytic paradigms. Many reported photothermal catalysts possess disordered crystallinity, irregular morphologies, and heterogeneous active sites, complicating precise control over solar‐to‐energy conversion efficiency and obscuring mechanistic understanding [14]. Consequently, rational design of advanced catalyst architectures that simultaneously deliver high photothermal efficiency and controllable reactivity, selectivity, and product upgrading is essential for advancing photothermal catalysis toward practical implementation [15].

Superlattices, defined by long‐range structural periodicity arising from atomic or molecular arrangements, or from the assembly of larger building blocks, exhibit a suite of distinctive physical and chemical properties [16, 17, 18]. Regardless of the fabrication pathways or the nature of inter‐unit coupling, such as covalent, metallic, or ligand‐mediated interactions, the defining feature of superlattices is their structural order at length scales comparable to those of photons, electrons, and phonons. Such structural precision directly gives rise to a quantum size effect, where dimensions approaching the electron wavelength induce energy‐level confinement and bandgap modulation, thereby strengthening light–matter interactions while regulating carrier transport. In parallel, the ordered and multiscale nature of superlattices increases the density and uniformity of surface active sites, enhancing catalytic activity. These attributes have already positioned superlattices as attractive platforms in photonics [19], magnetic materials [20], and optics [21]. Importantly, the quantum confinement and periodicity inherent to superlattices can impede electron and phonon transport, reducing energy dissipation and enabling highly efficient photo‐to‐heat conversion. Their ordered architectures also confer excellent thermal stability and durability, supporting stable operation under elevated temperatures and complex reaction environments. Therefore, superlattices provide an ideal platform for simultaneously optimizing photo‐to‐electron and photo‐to‐heat conversion, key requirements for efficient photothermal catalysis. Moreover, given that photothermal catalysis remains in an exploratory stage, with the relative contributions of photonic and thermal effects still under debate, the structural uniformity of superlattices offers a model system for reproducible experiments and rigorous elucidation of structure–property–performance relationships. This opens new opportunities to address longstanding challenges in photothermal catalysis, including enhanced reactivity, improved selectivity, and value‐added product upgrading through tandem or cascade reaction pathways.

**FIGURE 1:**
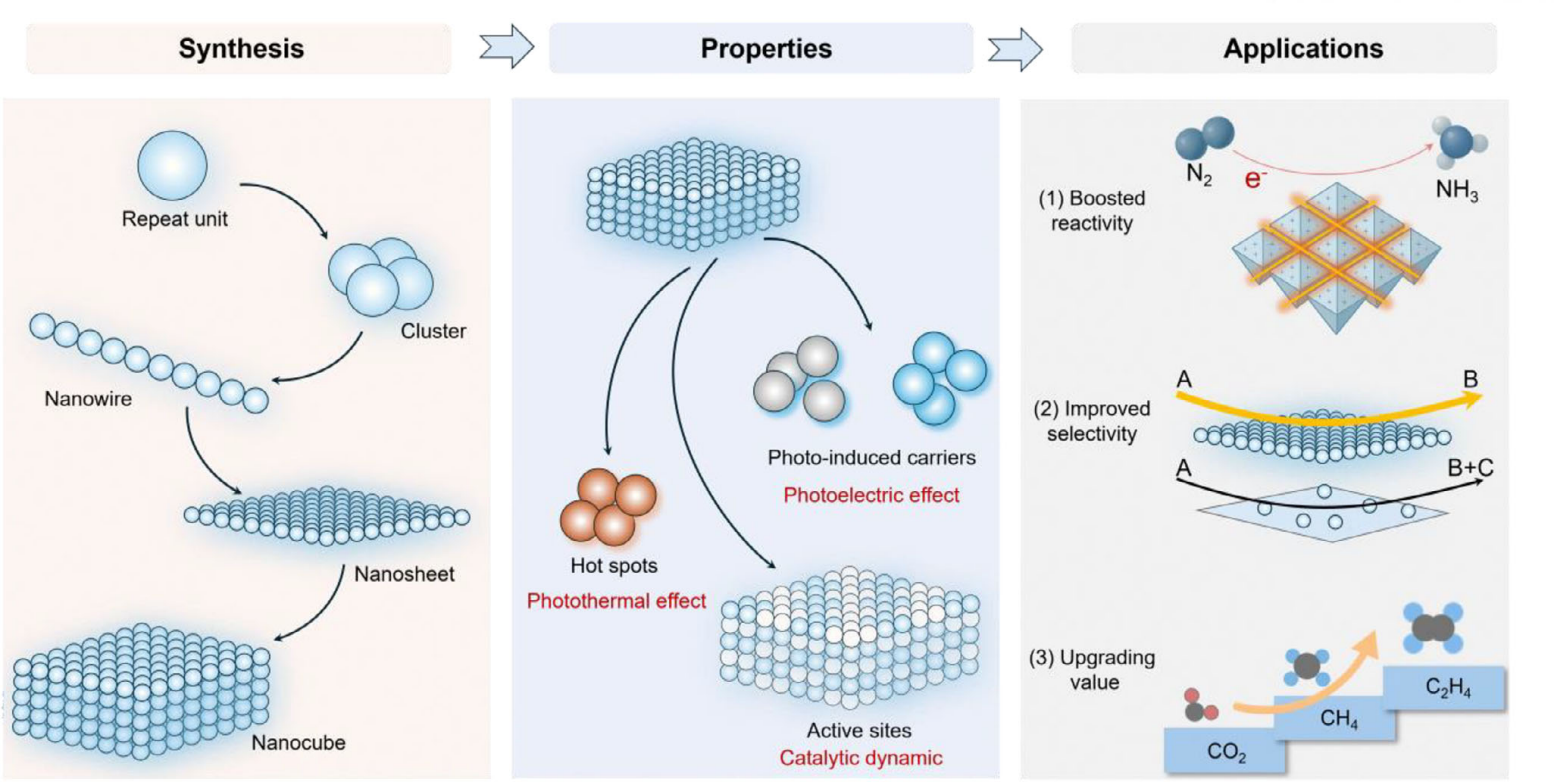
Schematic overview of superlattice‐driven advancement for photothermal catalysis.

In this review, we introduce superlattice‐based architectures as a unifying platform for capturing and managing solar energy in photothermal catalysis across a broad range of small‐molecule transformations (Figure 1). We will discuss the fundamental principles governing superlattice‐enabled photothermal processes, with particular emphasis on their roles in photoelectrochemical effects, photothermal energy conversion, and reaction thermodynamics. By critically summarizing recent advances and analyzing mechanistic insights, challenges, and emerging opportunities, we aim to provide a coherent framework for advancing superlattices in photothermal catalysis, ultimately guiding the development of more efficient, selective, and value‐oriented solar‐driven chemical transformations.

## 2 Fundamentals

### 2.1 Photothermal Catalysis

Photothermal catalysis, while sharing the same objectives as photocatalysis‐harnessing solar energy to drive chemical reactions‐ differs fundamentally in its reaction mechanism. In photothermal catalysis, a catalyst absorbs photons and converts solar energy into two key components: photo‐induced carriers and phonons. The photo‐induced carriers directly interact with the highest occupied molecular orbital (HOMO) and lowest unoccupied molecular orbital (LUMO) of the reactants, facilitating redox reactions. Simultaneously, the generated phonons lead to high localized temperatures, which help overcome energy barriers and accelerate reaction kinetics. The combined effects of these two components give photothermal catalysis an edge over traditional photocatalytic and thermocatalytic processes that rely on a single driving mechanism.

**FIGURE 2:**
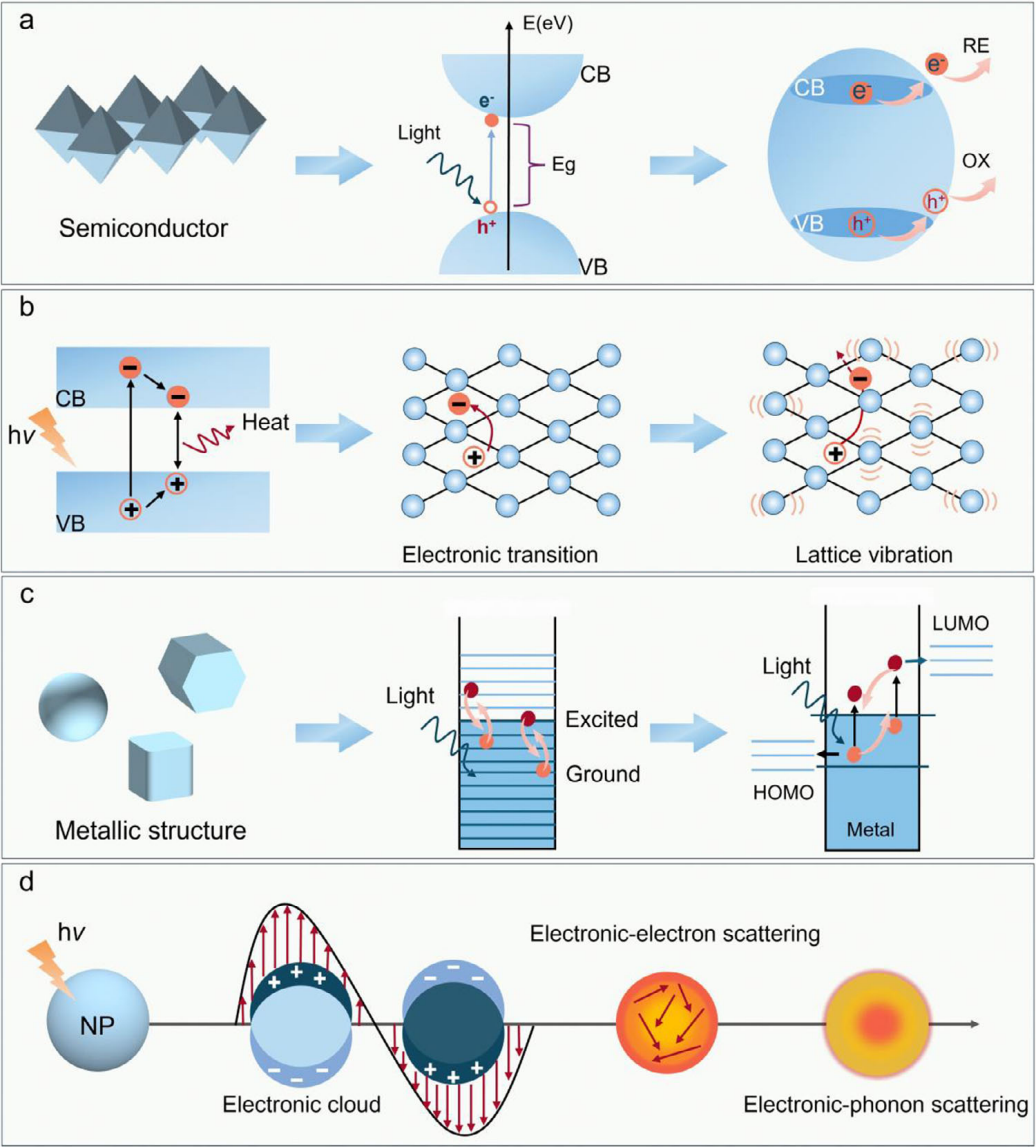
Mechanisms of photogenerated carrier dynamics and photothermal effects in semiconductors and metals. (a) Schematic illustration of photogenerated electron‐hole pairs in semiconductor materials participating in redox reactions. Reproduced with permission [23]. Copyright 2019, Advanced Materials. (b) Photothermal effects in semiconductors arising from electron‐hole recombination and lattice vibrations. Reproduced with permission [24]. Copyright 2023, Journal of Energy Chemistry. (c) Excited electrons in metals transferring to the HOMO‐LUMO levels of reactant molecules. Reproduced with permission [26]. Copyright 2014, Nano Today. (d) Photothermal effects in metals induced by electronic‐electron and electronic‐phonon scattering. Reproduced with permission [24]. Copyright 2023, Journal of Energy Chemistry.

A diverse range of materials exhibit photothermal properties, while the underlying mechanisms of their photothermal effects vary according to their intrinsic characteristics. For example, when a semiconductor is subjected to light irradiation, photons with energy equal to or exceeding the bandgap energy of the material will transfer their energy to electrons within the material. This energy transfer induces transitions of electrons from the valence band (VB) to the conduction band (CB), leaving holes in the VB [22]. The movement of these photo‐induced carriers is influenced by the electric field distribution within the semiconductor. Electrons will move towards the surface where they participate in reduction reactions (RE), while the holes migrate and engage in oxidation reactions (OX) [23] (Figure 2a). In the meantime, there is a strong Coulombic attraction between the electrons and holes, forcing them to be recombined when the absorbed energy is insufficient, thereby releasing heat in a non‐radiative manner. Furthermore, these excited‐state electrons can interact with phonons in the crystal and transfer energy to the phonons, leading them scattering. The phonon scattering results in atom and lattice vibrations, ultimately generating heat [24] (Figure 2b).

In contrast, for metallic materials under optical excitation, photons interact with electrons in the metal, transferring the energy to prompt the electrons' transition from their bound state to an elevated energy level [25]. Crucially, metals do not generate holes under light irradiation because electrons adhere to the Fermi‐Dirac distribution, resulting in a continuous distribution of energy levels without a distinct bandgap. Consequently, these excited‐state electrons can interact with reactant molecules by migrating to their HOMO or LUMO, thereby facilitating the cleavage or formation of chemical bonds and initiating a chemical reaction [26] (Figure 2c). Additionally, plasmonic states can be induced under optical excitation. In these states, there are intense interactions among electrons and ions, including electron‐electron, electron‐ion, and ion‐ion collisions. These interactions cause a significant energy redistribution, which raises the temperature of both electrons and ions, thereby generating heat [24] (Figure 2d).

Despite current photothermal catalysts show promising performance in reducing reaction barriers, enhancing built‐in electric fields (BIEF), and accelerating transport of photo‐induced carriers, they suffer from significant limitations. Specifically, achieving exceptional photo‐to‐electron and photo‐to‐thermal efficiencies within a single material is still a formidable challenge, which hinders the realization of optimal photothermal synergetic effects. Furthermore, the mechanisms of individual electron‐generated and heat‐generated processes are relatively understood, and the synergistic mechanism of the photothermal effect is not yet fully elucidated. Consequently, there is a pressing need to develop advanced photothermal catalysts to address these shortcomings.

### 2.2 Superlattice Materials

In the realm of photothermal catalysis, superlattices emerge as innovative materials, promising to address existing challenges and optimize efficiency due to their periodic arrangements and highly ordered structures. Superlattices are a type of crystalline structure with a periodic arrangement, composed of various crystal units combined in a specific geometric pattern in space to form a highly ordered structure with periodic characteristics. According to the principle of minimum energy, when different crystal phases or orientations assemble, an energy difference arises at the interface between them. To reduce this interfacial energy, the crystal units tend to arrange in an ordered pattern, ultimately forming superlattices. The binding between these crystal units is typically governed by van der Waals forces [27], charge interactions [28], surface tension [29], and solvent effects [30].

The construction of such periodic architectures generally relies on two complementary strategies: top‐down fabrication and bottom‐up assembly. The top‐down approach focuses on the deliberate reconstruction of pre‐existing materials. In this strategy, bulk crystals, thin films, or two‐dimensional layers are first prepared and subsequently reconfigured into superlattice structures with controlled periodicity via techniques such as mechanical exfoliation, dry or wet transfer, angle‐controlled stacking, and template‐confined fabrication [31, 32]. In contrast, the bottom‐up approach starts from fundamental building units, including atoms, molecules, clusters, or nanocrystals, and progressively assembles them into long‐range ordered structures through epitaxial growth, self‐assembly, or in situ reactions.

Conventional semiconductor and oxide superlattices are typically fabricated by layer‐by‐layer deposition methods, such as metal‐organic chemical vapor deposition and atomic layer deposition, whereas colloidal or nanocrystal superlattices are more commonly formed through solvent‐evaporation‐induced assembly, interfacial assembly, and ligand‐mediated assembly [33, 34]. Overall, the top‐down route offers precise structural control and design flexibility, making it particularly suitable for constructing artificial superlattices with well‐defined interfaces and tunable twist angles or spatial arrangements. By comparison, the bottom‐up route is more advantageous for large‐area fabrication, scalable production, and controllable construction of complex multicomponent systems.

**FIGURE 3:**
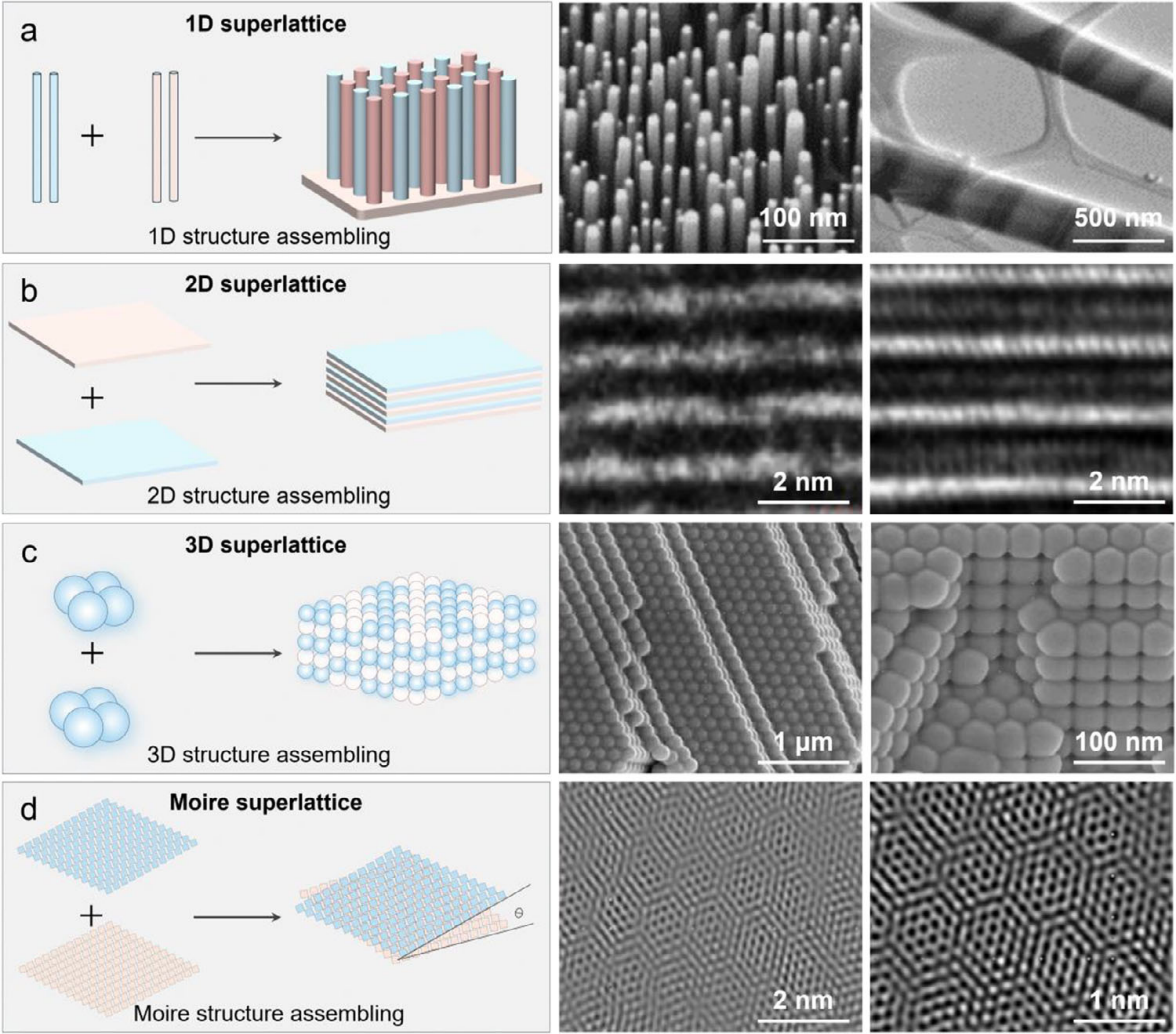
Representative types of superlattice constructed from various nanostructures. (a) 1D superlattice with observed nanorod structure. Reproduced with permission [34]. Copyright 2002, Nano Letters. (b) 2D superlattice with observed nanosheet structure. Reproduced with permission [37]. Copyright 2021, Chem. (c) 3D superlattice with observed nanosphere structure. Reproduced with permission [38]. Copyright 2006, Macromolecular Chemistry and Physics. (d) Moiré superlattices with observed twisted nanosheet structure. Reproduced with permission [39]. Copyright 2021, Nature Communications. Each panel includes a schematic illustration of the ordered assembly and its corresponding microscopy image (SEM or TEM, as indicated).

Based on their structural dimensionality, superlattices can be classified as one‐dimensional (1D), two‐dimensional (2D), and three‐dimensional (3D) materials. 1D superlattices consist of linearly arranged lattice units, always manifesting as nanowires or nanotubes [35] (Figure 3a). A representative example is 1D gallium arsenide (GaAs)/gallium phosphide (GaP) superlattice nanowires, which are fabricated by laser‐assisted catalytic growth techniques [36]. The resultant defect‐free atomic junctions hold a significant promise for applications in photonics and electronics. 2D superlattices, typically composed of planarly arranged lattice units, are often found in thin‐layer structures like graphene (Figure 3b). These monolayers, with their highly uniform thickness, can be assembled into alternating hybrid superlattices, generating multifunctional artificial superlattices with tunable chemical compositions and structural periodicities [37]. The distinctive layer‐stacking nanostructure in these materials induces various unique effects, such as superior catalytic activity by providing more active sites for reactions and tunable thermal conductivity for efficient thermal management. In contrast, 3D superlattices exhibit more stereoscopic crystal structures (Figure 3c). For instance, 3D superlattices can be formed by the precise ordering of quantum dot nanocrystals and demonstrate excellent responses to magnetic, electric, and optical stimuli with finely tuneable properties [38].

Notably, in 2D layered superlattices, slight differences and interactions between the periodic structures can give rise to interference effects, leading to the formation of periodic Moiré superlattices [39] (Figure 3d). These differences often arise from lattice mismatch [40] (such as variations in lattice constants or orientations) and geometric mismatches [41] (such as rotation and translation). Additionally, the presence of lattice defects and heterojunctions can promote the formation of Moiré superlattices. The locally enhanced interlayer coupling in these structures leads to changes in the electronic band structure and optoelectronic properties. As such, Moiré superlattices with modulated physical properties show promising use in optoelectronics, photocatalysis, and practical environmental purification [42].

Evidently, superlattices possess unique structural characteristics, and the foremost challenge lies in the precise arrangement of constituent particles according to a predefined pattern. Even if such structural control can be achieved at a small scale, scalable construction remains a formidable obstacle. Moreover, beyond the realization of ordered architectures, the fine‐tuning of morphology, such as selective facet exposure and the assembly of particles with irregular or complex shapes, to enhance optical and photoelectrochemical properties, is still largely unexplored. It is also noteworthy that, while 2D and 3D architectures currently dominate experimental studies in photothermal catalysis, 1D and Moiré classes are increasingly attracting attention due to their unique ability to modulate carrier and phonon transport. More critically, the photothermal conversion mechanisms of such structures have not been fully elucidated, posing significant limitations for rational design and performance optimization. Therefore, the following section aims to provide an in‐depth analysis of the relationship between superlattice structures and photothermal catalysis, with a particular focus on structural order influences on photo‐to‐electricity conversion, photo‐to‐thermal conversion, energy transfer, and the coupling with reaction thermodynamics, thereby offering theoretical insights and practical guidance for the development of efficient and controllable photothermal catalytic systems.

## 3 Distinctions of Superlattices in Photothermal Catalysis

Due to their distinctively repetitive structures, superlattices exhibit exceptional potential for addressing the complex challenges currently encountered in photothermal catalysis, facilitating highly efficient synergy between photoelectrochemical and photothermal effects. These architectures not only optimize light absorption and conversion but also impart notable advantages in both photoelectrochemical and photothermal performances. Furthermore, their structural adaptability allows for meticulous tailoring to accommodate various catalytic reactions, thereby significantly enhancing catalytic kinetics. In this section, a thorough analysis of the advanced photoelectrochemical, photothermal, and catalytic kinetics capabilities of superlattices will be presented, underscoring their profound potential within the domain of photothermal catalysis. Also, the unique structural characteristics of superlattices in facilitating effective coupling of photoelectrochemical and photothermal phenomena will be discussed, along with targeted structural optimization and functional tuning for elevating their catalytic performance. These insights will underscore the pivotal role of superlattices in the evolution of photothermal catalytic technologies and their extensive prospects for future applications.

### 3.1 Unique Photoelectrochemical Effect

**FIGURE 4:**
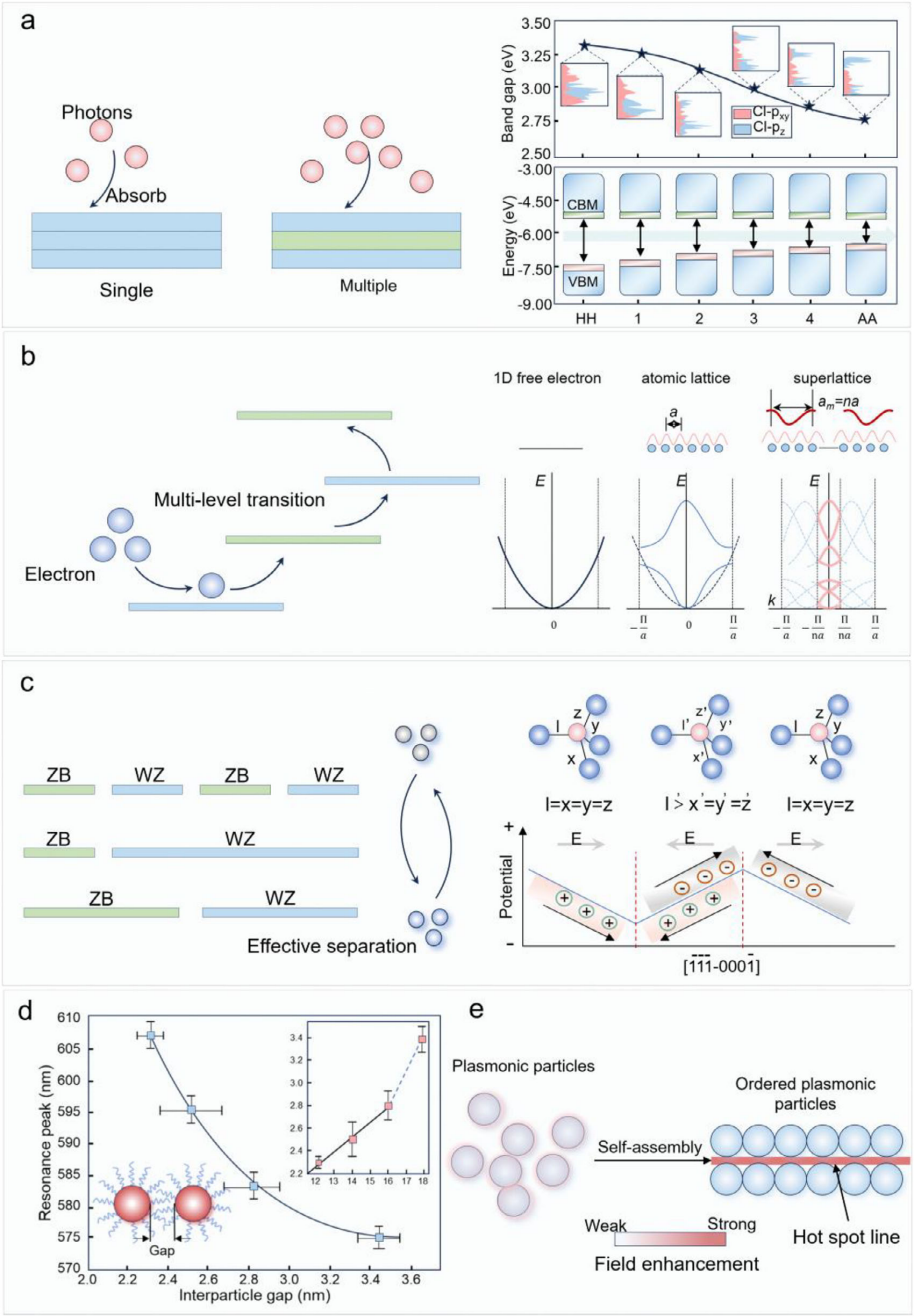
(a) Comparison of light absorption properties of different materials. Reproduced with permission [44]. Copyright 2019, Nature Communications. (b) Multi‐level transition of electrons. Reproduced with permission [45]. Copyright 2023, Advanced Materials. (c) Saw‐tooth‐like potential profile and the built‐in electronic field. Reproduced with permission [46]. Copyright 2017, Nano Energy. (d) Exponential dependence of the resonance peak position on the interparticle gap. Reproduced with permission [51]. Copyright 2008, Journal of the American Chemical Society. (e) Schematic depiction of the edge‐edge coupling. Reproduced with permission [52]. Copyright 2023, Angewandte Chemie International Edition.

Photo‐to‐electron conversion is a crucial process in photothermal catalytic reactions, where EHCs generated after photon absorption can overcome thermodynamic barriers and directly participate in solar fuels production. In semiconductor‐based photocatalytic processes, EHCs are excited through energy band transition, meaning the band structure plays a critical role in determining photo‐to‐electron efficiency and charge dynamics. However, traditional semiconductor materials often exhibit a wide bandgap, restricting their ability to absorb light across a broad spectral range. Additionally, these materials are often plagued by intrinsic structural inhomogeneities, resulting in defect states and impurity levels. These imperfections can function as active centers for the recombination of photo‐induced carriers, thereby impeding the generation of EHCs. In contrast, semiconductor superlattices, composed of multiple repetitive periodic units, offer both electronic and photonic advantages [43]. Electronically, inter‐unit coupling in the superlattice can upshift the valence band maximum (VBM) by enhancing anti‐bonding interactions across adjacent layers, while maintaining a stable conduction band minimum (CBM). This effectively narrows the bandgap (Figure 4a), enabling absorption across a broader solar spectrum [44]. Structurally, the long‐range periodicity introduces photonic effects that conventional materials lack. The repeating units induce Bragg scattering and interference patterns, which extend photon residence time through multiple internal reflections and constructive interference.

Beyond enhancing light absorption, the periodic potential of superlattices further modifies electronic transitions by restructuring the band landscape. Unlike conventional semiconductors that offer single‐band transitions, superlattice semiconductors exhibit multiple bandgaps and distributed energy levels, enabling multi‐level electron transitions. Free electrons typically follow a parabolic dispersion under a constant 1D potential [45] (Figure 4b), but the band structure folds relative to the Brillouin zone (BZ) boundaries when a lattice periodic potential is introduced. In well‐defined epitaxial systems, this folding has been demonstrated to generate new bandgaps and reorganize the band organization, a mechanism that is anticipated to similarly govern electronic transitions in more complex superlattice catalysts. Moreover, the introduction of a long‐period superlattice potential with Moiré periodicity, a_m_ = na (n≠1) (where a represents the lattice constant, m is the periodic number, and n is the multiple), further modifies the band structure by folding it into a Moiré BZ, creating replica bands and restricting electron behavior. This arrangement allows for photo‐generated electrons to transition across multiple energy levels, greatly reducing the likelihood of recombination with holes. Additionally, superlattices can be arranged into different stacking modes, resembling heterojunctions, which guide the flow of electrons and holes into separate components, further reducing recombination rates and extending the lifetime of the charge carriers.

Notably, successive diffusion and drift of charge carriers within a material result in an interfacial BIEF, which plays a crucial role in the dynamics of photo‐generated electron‐hole pairs. This field significantly accelerates the rapid transfer of EHCs and minimizes their loss during the transfer process. In polarized layered architectures, the BIEF can be significantly amplified. When such interfaces are periodically stacked, the local potential differences can accumulate constructively, leading to an enhanced overall driving force for charge separation, as demonstrated in various heterostructured systems. Furthermore, when superlattices are composed of materials with differing electronegativity, such as II–VI and III–V compounds, the charge separation and polarization become more pronounced, increasing the bilateral potential difference and further strengthening the BIEF. For instance, a zincblende‐wurtzite (ZB‐WZ) superlattice structure forms a sawtooth‐like potential distribution within the superlattice cell due to spontaneous polarization [46] (Figure 4c). This causes electrons in the CBM and holes in the VBM alternately accumulate around the ZB/WZ heterojunction interface, rather than being pulled into the interiors of adjacent ZB and WZ segments. Therefore, this configuration leads to a strong BIEF to significantly promote the efficient transfer of photo‐generated charges, effectively leveraging the unique electronic properties of the superlattice to optimize charge dynamics.

Interestingly, the polarization phenomenon can be amplified by a strain effect [47], which often arises during the fabrication of superlattices due to the differences in lattice periods and constants between materials, as well as interface effects. Strain not only impacts the electronic properties but also influences the distribution of active catalytic sites. In a photocatalytic water splitting process, for example, the favorable sites for the hydrogen evolution reaction (HER) are typically located at edges, polycrystalline interfaces, and a strained metal phase, where ΔG_H_ values approach zero, indicating higher catalytic efficiency. Therefore, strain effects can be engineered to modulate the active sites in these regions, optimizing the performance and efficiency of catalytic systems [48].

On the other hand, EHCs can also be excited in nano‐sized metals via a surface plasmon resonance (SPR) effect. When the energy of incident photons resonates with the SPR frequency of a metal, the photon energy can be transferred to the free electrons in the metal, thereby exciting surface plasmons and achieving photon absorption. However, the SPR frequency is highly dependent on both the particle size and electron density of the metal. In current metal‐based photothermal catalysts, issues such as uneven particle size and spatial dispersion often lead to suboptimal resonance conditions. This misalignment can result in either reduced resonance intensity or a spectral response outside the optimal range, thereby limiting the efficiency of plasmonic excitation for generating EHCs. These challenges ultimately restrict the overall effectiveness of a metal catalyst in photothermal applications.

In contrast, metallic superlattices, while achieving photon absorption through a similar SPR mechanism, also benefit from their unique periodic structure. The ordered arrangement of unit cells in a metallic superlattice can induce collective effects to significantly influence plasmonic resonance. By precisely controlling the arrangement of these unit cells into larger, ordered domains, it becomes possible to fine‐tune the coupling between individual building blocks, which can further enhance the plasmonic resonance effect and improve photo‐to‐electron conversion efficiency [49, 50]. Studies have shown that the interparticle spacing plays a key role in determining the localized surface plasmon resonance (LSPR) in superlattice metallic materials. As the gap between particles increases, the resonance peak decreases, displaying an exponential correlation [51] (Figure 4d).

Additionally, the coupling between neighbouring nanoparticles (NPs) can lead to the formation of pronounced plasmonic hotspots, particularly at the particle edges. These hotspots arise from collective surface plasmon oscillations, which significantly amplify the local electromagnetic field. The enhanced field strength in these regions further increases the photo‐to‐electron conversion efficiency by raising the density of EHCs in localized areas [52] (Figure 4e). The presence of strong plasmonic hotspots not only intensifies the light‐matter interaction but also facilitates more efficient energy transfer processes, leading to substantial improvements in catalytic performance and photothermal response. This phenomenon underscores the importance of precise nanoparticle design and spatial arrangement in optimizing the plasmonic properties for advanced photothermal applications.

Superlattice structures, whether a semiconductor or metal, are engineered to optimize specific photoelectrochemical properties and achieve unique photoelectrochemical effects. These structures employ ordered, periodic layers to modulate electron behavior, thereby enhancing the efficiency of photo‐to‐electricity energy conversion. Despite their considerable advantages in boosting photoelectrochemical conversion efficiencies, superlattices also present notable challenges that merit attention. For instance, while strain effects can enhance carrier mobility, excessive strain accumulation may compromise the structural stability of the interior. One potential solution could involve integrating structurally stable substrates like graphene to fortify the overall ordered structure. Therefore, future research must not only leverage the strengths of superlattices but also uncover and address the underlying challenges that may limit their application. This balanced approach will be crucial for advancing superlattice technologies in photoelectrochemical applications, ensuring both structural integrity and performance enhancement.

### 3.2 Strengthened Photothermal Effect

Photo‐to‐thermal conversion is another vital process in photothermal catalysis, enabling the use of ultraviolet, visible, and infrared light to achieve full‐spectrum sunlight harvesting. This conversion occurs mainly through two pathways: non‐radiative recombination of photo‐generated electron‐hole pairs and interaction of photons with phonons or electrons within a material. In semiconductors, the interaction between photons and phonons induces lattice vibrations, while in metallic materials, the interaction between photons and electrons or ions triggers electron‐electron, ion‐ion, and electron‐ion oscillations, thus generating heat [53].

However, achieving high photo‐to‐thermal conversion efficiency remains challenging due to limited light absorption, intrinsic energy dissipation during conversion, and suboptimal material architectures in restricting efficient heat localization. In particular, the thermal energy generated at localized hot spots tends to dissipate rapidly via conduction, convection, and radiation, undermining the overall thermal enhancement. Thus, effective regulation of the generated heat to maximize the utilization of photothermal energy is of vital importance in photothermal catalysis. The solution lies in designing advanced thermal barrier layers or heat‐absorbing layers within photothermal catalysts to improve conversion efficiency and reduce heat loss. Compared to traditional nanomaterials, superlattices offer better performance in photothermal conversion and heat storage.

**FIGURE 5:**
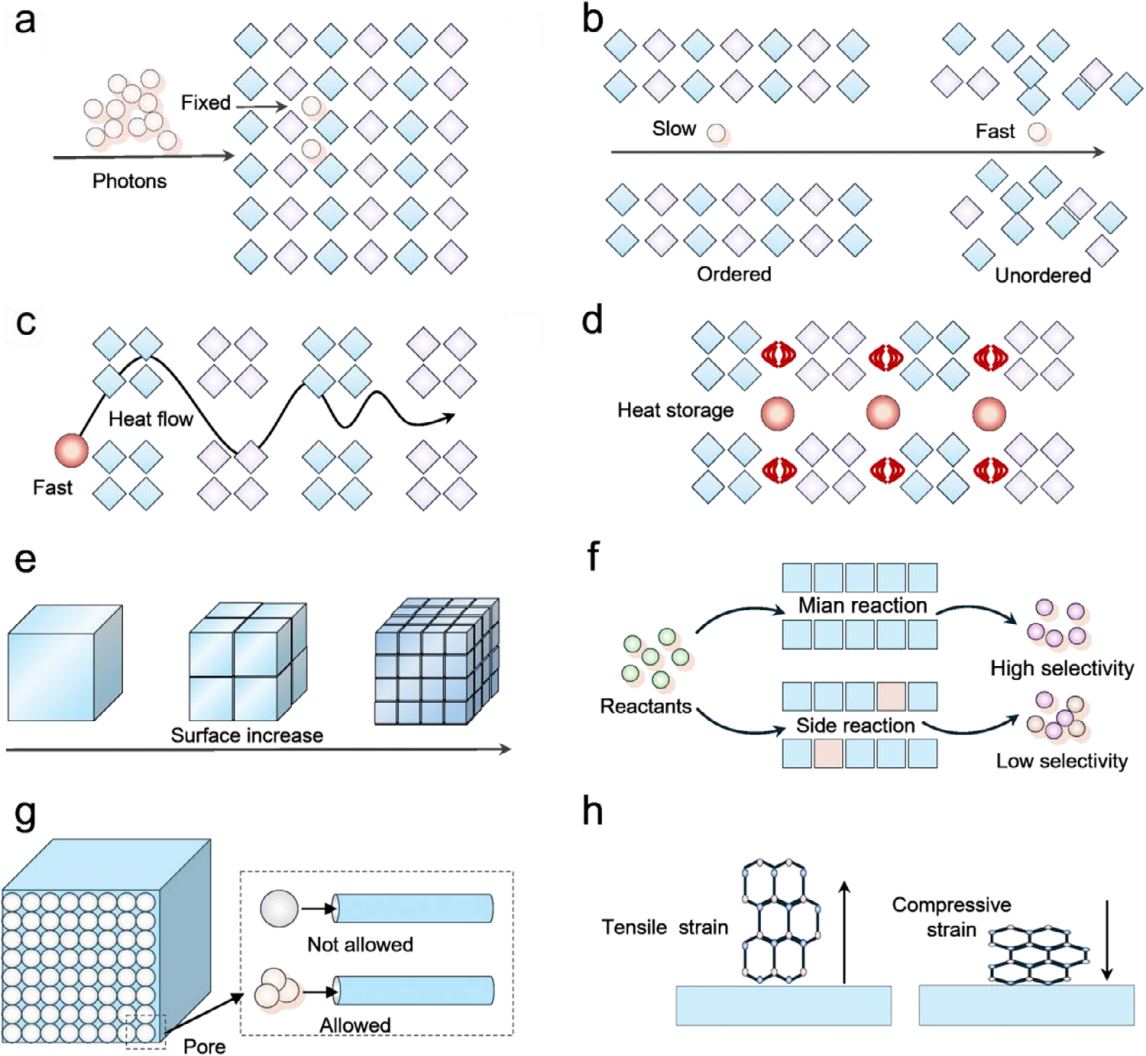
(a) PBG effect, (b) SP effect of a semiconductor superlattice. (c) Reduced phonon group velocity via interaction between repeating units. (d) Improving heat storage capacity by coupling between repeated units. (e) Larger surface areas in superlattice materials. f) Higher product selectivity in superlattices. (g) Arrangeable active sites in repeating units. (h) Two ways of strain effect: tensile strain and compressive strain.

In semiconductor superlattices, photon propagation is constrained by the band structure, resulting in a phenomenon known as the photonic bandgap (PBG) effect, where photons within certain energy ranges are unable to propagate [54] (Figure 5a). The PBG effect originates from Bragg diffraction and scattering, and its intensity diminishes as the number of photonic crystal (PC) layers decreases. By adjusting the number of layers in superlattices, the PBG intensity can be strengthened, improving photon absorption and broadening the absorption spectrum, thus boosting photothermal conversion efficiency. For instance, it was found that the PBG effect could enhance the absorption in the near‐infrared region when constructing PC films by integrating CdS and graphene oxide nanosheets in a superlattice structure [55].

Additionally, when the wavelength of incident light approaches the edge of the PBG in a PC superlattice, the group velocity of photons is significantly reduced, giving rise to the slow photon (SP) effect [56] (Figure 5b). This effect extends the optical path length effectively and increases the interaction time between photons and the active medium, thereby enhancing light absorption and its conversion into heat. In TiO_2_ photonic crystals, the SP effect has been shown to operate efficiently within a 400–800 nm range, where photon slowing is particularly pronounced. Structural modulation, such as tuning the layer thickness or periodicity, further intensifies localized photothermal effects by reinforcing photon confinement [57]. These findings underscore the critical role of the SP effect in improving photo‐to‐thermal conversion efficiency and demonstrate the potential of photonic superlattice design for advanced photothermal applications.

For metallic superlattices, photons can be absorbed and converted into heat through the LSPR effect [58]. The periodic arrangement of metallic units induces interparticle coupling across different repeating layers, enhancing the intensity and spatial extension of localized surface plasmons [59, 60, 61]. This effect has been experimentally validated in systems such as periodic gold nanocuboids, where a reduced gap distance between adjacent nanocuboids leads to stronger plasmonic coupling and a more pronounced photothermal response [62]. Furthermore, under optical excitation, collective oscillation modes arising from these couplings amplify local electromagnetic fields and promote efficient heat generation, thereby improving the overall photothermal conversion efficiency [63].

While efficient heat generation is essential, the ability to retain and confine the generated heat locally is equally critical for maximizing photothermal catalytic performance. The periodic structure of superlattices contributes to effective thermal confinement. From a solid‐state physics perspective, enhanced phonon scattering at the interfaces between periodically arranged units can significantly impede phonon transport and suppress thermal conduction. This interfacial scattering effect is hypothesized to facilitate localized heat retention within the catalytic motifs, leading to a reduced effective thermal conductivity compared to bulk counterparts. Furthermore, variations in the composition or spatial configuration of the repeating units can modulate phonon dispersion relations, altering vibrational frequencies and modes and thereby reducing phonon group velocities (Figure 5c). Through precise control over interfacial spacing and structural arrangement, thermal transport can be effectively regulated [64, 65]. For example, the thermal conductivity of Si/Ge superlattice nanowires has been reported to decrease by an order of magnitude compared to homogeneous nanowires, primarily due to strong phonon‐interface interactions that significantly slow down phonon propagation [66].

Beyond suppressing thermal conductivity, interfacial engineering in hybrid superlattices can also enhance effective heat capacity and thermal retention by altering local atomic environments and phonon dynamics. When superlattices incorporate dissimilar constituent materials, the resulting interfaces significantly modify the local structure, giving rise to notable interfacial effects. These effects can alter the phonon density of states by introducing localized interfacial vibrational modes, thereby modifying phonon distribution and increasing the effective thermal capacity of the material (Figure 5d). For instance, TiO_2_‐ and ZnO‐based hybrid superlattice structures, which feature periodic organic layers between inorganic components, exhibit an enhanced heat capacity after annealing, attributed to higher interfacial density and stronger interfacial bonding. The closer integration of materials at the interface magnifies these interfacial effects, contributing to a more efficient thermal storage capacity and improved thermal management within the superlattice structures [67]. Importantly, this improvement is not exclusive to specific materials, and it can be extended to other hybrid systems with the interfacial effects to optimize thermal properties. By fine‐tuning the composition and arrangement of the constituent materials, a higher heat storage capacity and thermal efficiency can be achieved.

Superlattices, with their ordered architectures, not only enhance light absorption and extend photon scattering pathways, but also suppress phonon transport, thereby improving photothermal conversion efficiency. This makes them highly attractive for solar‐to‐thermal energy conversion. However, this thermal confinement effect presents a trade‐off: while it promotes internal heat retention, it can also limit heat flow to active catalytic sites, reducing overall reaction efficiency. Additionally, superlattices composed of dissimilar materials may accumulate internal stresses under high temperatures due to mismatched thermal expansion coefficients, potentially compromising structural integrity. To mitigate these challenges, rational design strategies should prioritize the selection of components with compatible thermal expansion properties or the incorporation of interfacial buffer layers to alleviate stress accumulation. Such approaches are essential to achieving a balanced optimization of photothermal efficiency, catalytic performance, and structural durability.

### 3.3 Boosted Reaction Dynamics

In addition to electrochemical and thermal considerations, understanding reaction dynamics is critical to optimizing the overall performance of photothermal catalysis. Among the factors influencing reaction kinetics, the spatial distribution and structural characteristics of active sites are particularly important. These surface regions serve as catalytic centers, where energy absorption, charge transfer, and molecular activation converge to directly influence the rate and progression of reactions. In conventional photothermal catalysts, the number and accessibility of active sites are often difficult to control due to particle aggregation and morphological changes during synthesis processes such as deposition or sintering. This can result in inefficient utilization of catalytic surfaces and unstable kinetic behavior under light‐driven conditions. In contrast, superlattices provide a highly organized framework with large specific surface areas (Figure 5e), enabling the deliberate design of accessible and uniformly distributed active sites. Such a structural control ensures consistent catalyst‐reactant contact, promotes efficient energy transfer at the reaction interfaces, and supports stable and accelerated reaction kinetics [68].

The tunable periodic structure of superlattices enables the design of repeatable units with highly consistent active sites, which can be systematically arranged to ensure synchronized and cooperative reaction processes. Therefore, the structural homogeneity of superlattices provides a uniform and predictable reaction environment to suppress side reactions and enhance both product selectivity and purity (Figure 5f). In parallel, their ordered electronic structures help maintain consistent charge distribution across active sites, forming a robust basis for the precise electronic control of catalytic behavior. Beyond the uniformity of active sites, some superlattices also exhibit intricately ordered pore architectures, which restrict the diffusion and adsorption of reactant molecules [69] (Figure 5g). This steric and polarity‐based confinement enables size‐selective and polarity‐selective transformations, thereby minimizing competitive adsorption and further improving both selectivity and catalytic efficiency [70, 71].

Beyond structural uniformity and pore confinement, another distinctive advantage of superlattices lies in their ability to generate controllable strain fields. When composed of alternating layers with different lattice constants, superlattices inherently induce periodic interfacial strain [72] (Figure 5h). This periodic interfacial strain is believed to modulate the local atomic environment and alter the electronic structure of surface atoms. Such electronic modifications are closely associated with shifts in the adsorption energies and stability of reaction intermediates, providing a powerful means to regulate catalytic pathways [73]. As a result, strain engineering can suppress premature desorption or undesirable conversion of intermediates, stabilize favorable reaction pathways, and improve overall catalytic efficiency. However, despite these multifaceted advantages in photoelectrochemical, thermal conversion, and reaction control, integrating them into a single superlattice material remains a formidable challenge. Effective implementation requires precise control over both material composition and structural architecture. First, drawing from existing experiences with photothermal catalysts, the construction of core–shell structures [74, 75] and arranging them in orderly arrays ensure concentrated heat management while meeting superior photovoltaic conversion efficiencies. The coupling of different units within these structures may also introduce new advantages. Second, incorporating broadband‐absorbing substrates, such as black materials [76, 77], loaded with spatially organized metallic NPs not only maximizes photothermal effects but also facilitates the generation of more hot electrons. Additionally, advanced computational models [78, 79] offer predictive insights into the thermodynamic and structural compatibility of heterogeneous components, aiding in the design of stable and functional superlattices. With ongoing advances in materials science and nanotechnology, future superlattices are expected not only to further optimize photothermal conversion efficiencies but also to enable sophisticated reaction control in solar fuel generation and environmental catalysis.

**FIGURE 6:**
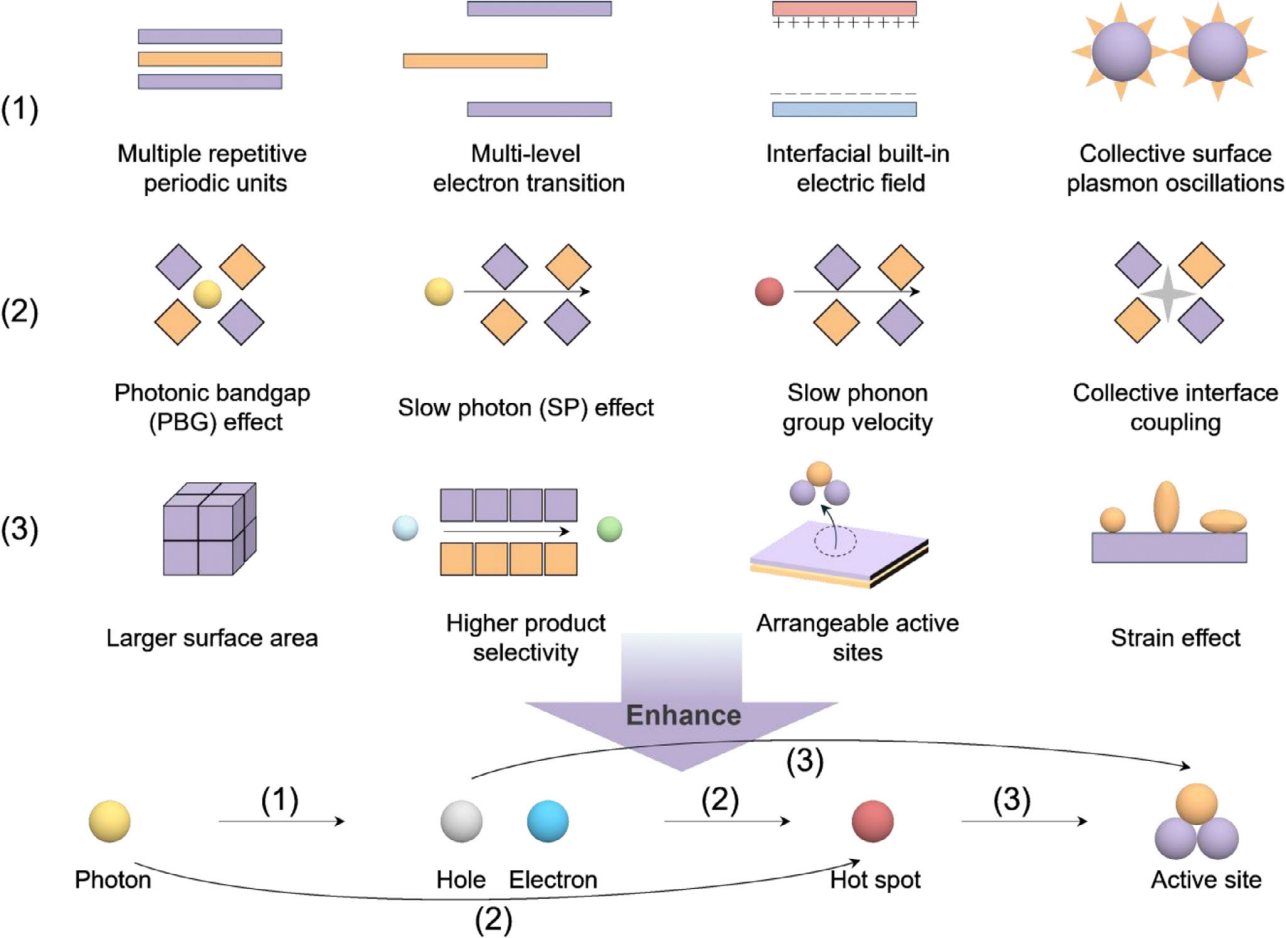
Schematic illustration of the enhanced photothermal catalytic performance via superlattices. The process includes: (1) Photons excite electrons and holes, enabling charge carrier generation; (2) Photons are directly converted into heat, and nonradiative recombination of electrons and holes further releases thermal energy; (3) Resulting photothermal effects, including localized hot spots and energetic charge carriers, activate surface catalytic reactions and improve overall catalytic efficiency.

In conclusion, superlattices, with their periodic and tunable architectures, contribute across all stages of a photothermal catalytic process, ranging from light harvesting to thermal conversion and reaction kinetics. In a photochemical process, the long‐range periodicity enables precise bandgap modulation and enhances light absorption via photonic effects, while the repetitive inter‐unit coupling facilitates efficient charge separation by strengthening internal electric fields. In the photothermal stage, the ordered lattice symmetry supports the PBG and SP effects to boots light‐to‐heat conversion, while interfacial phonon scattering between repeating units reduces thermal conductivity and enhances heat retention. At the reaction level, superlattices accelerate kinetics and improve selectivity by spatially organizing active sites, engineering periodic surface strain, and providing hierarchical porosity. More importantly, these functional stages are not independent but are strongly coupled. While intrinsic factors, such as composition and defects chemistry, establish the catalytic foundation, the superlattice architecture acts as a structural amplifier that simultaneously regulates light management, charge dynamics, and thermal behavior. These interdependencies underscore the necessity of moving beyond isolated optimization toward integrated design strategies. In this context, superlattices represent not only a structurally coherent platform but also a next‐generation catalyst framework for solar‐driven chemical transformations (Figure 6).

To fully realize the promise of integrated photothermal design, it is essential to acknowledge and address the structural trade‐offs inherent to superlattice architectures. While highly ordered architectures are beneficial for directional charge transport, it may also cause photogenerated electrons to migrate too rapidly across active interfaces. This excessive carrier mobility can reduce the residence time of electrons at catalytic sites, thereby limiting the probability of desired surface reactions and lowering overall reaction efficiency. Also, interfacial effects within superlattice structures can suppress phonon group velocities, and this divergence in carrier and lattice dynamics may weaken electron–phonon interactions. In particular, rapid electronic delocalization combined with reduced phonon group velocities could diminish the efficiency of non‐radiative relaxation processes, thereby limiting photothermal energy conversion. Therefore, how to fully exploit the advantages of superlattices while mitigating their inherent drawbacks remains a challenge. It may be possible to construct ordered/disordered hybrid structures to preserve the structural advantages of superlattices while addressing their drawbacks through enhanced phonon scattering and improved carrier and mass transport regulation.

## 4 Techniques for Mechanistic Investigations

Superlattices offer distinct advantages in photo‐to‐electron conversion, photo‐to‐thermal processes, and reaction dynamics. However, their application in photothermal catalysis is still in its infancy, necessitating in‐depth microscopic investigations to resolve unit‐level arrangements. Although periodicity is a defining feature of superlattices, the precise nanoscale arrangement of repeating units and the underlying factors governing their assembly are still not fully understood. Additionally, charge transfer mechanisms within superlattices, particularly those occurring on picosecond to femtosecond timescales, remain insufficiently explored. Understanding the coupling between adjacent units and how it governs carrier migration is essential for achieving precise control over charge dynamics. Moreover, the transfer of thermal energy within superlattices and how localized variations in thermal energy influence the material's properties, are keys for optimizing the photo‐to‐thermal conversion efficiency in photothermal catalysis.

**FIGURE 7:**
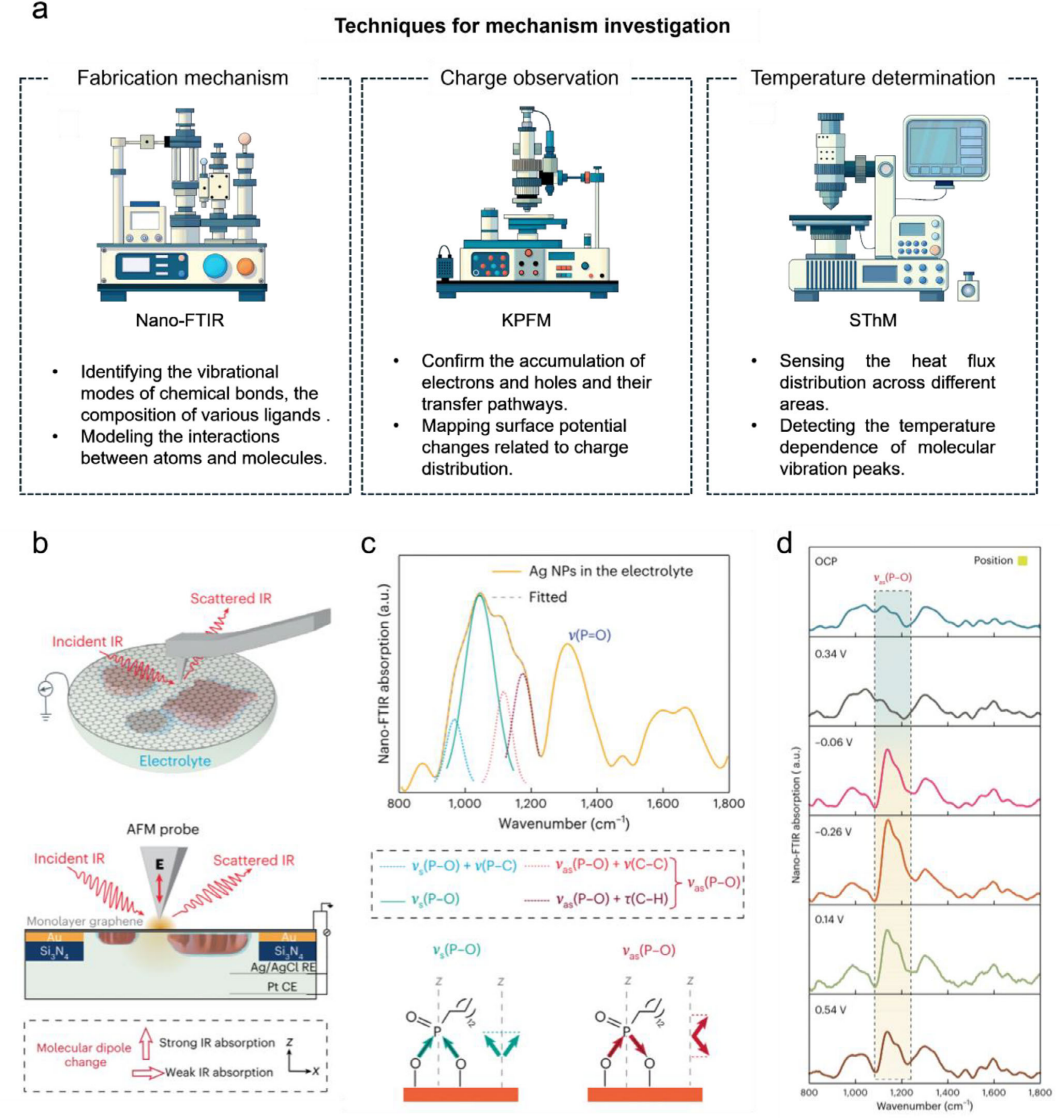
(a) Advanced techniques for mechanistic investigations in photothermal catalysis. (b) Schematic of nano‐FTIR experiment [88]. (c) Nano‐FTIR spectra of Ag NPs within a cell filled with an electrolyte under an open‐circuit condition [88]. (d) Stacked nano‐FTIR spectra under stepped bias collected at the specific position [88]. Reproduced with permission [88]. Copyright 2024, Nature Catalysis.

To address these challenges, we propose a research framework that integrates emerging advanced characterization technologies to probe the nanoscale properties of superlattices (Figure 7a). For example, infrared nanospectroscopy (Nano‐FTIR) can be used to identify the vibrational modes of chemical bonds and the composition of various ligands and provide insights into the synthesis process of superlattices. Additionally, using artificial intelligence can assist to model the interactions between atoms and molecules, helping in designing optimal superlattice structures. Building on this, surface photovoltage microscopy (SPVM) technology can confirm the accumulation of electrons and holes and their transfer pathways, Kelvin probe force microscopy (KPFM) maps surface potential changes related to charge distribution, scanning thermal technology (SThM) detects the heat flux distribution across different areas, and tip‐enhanced Raman spectroscopy (TERS) measures the temperature dependence of molecular vibration peaks. Together, these tools advance a comprehensive understanding of photothermal mechanisms and optimize material design.

### 4.1 Fabrication Mechanism

Superlattices exhibit exceptional properties arising from their unique periodic nanostructures, which can be engineered by adjusting the constituent materials, their spatial arrangements, and inter‐unit distances. However, understanding the microscopic interactions and combinations of these repeated units remains a challenge. Currently, three main strategies are commonly employed for superlattice self‐assembly: (1) solid‐substrate assembly [80], (2) liquid–interface assembly [81], and (3) confinement‐guided assembly [82]. The driving forces behind these methods vary depending on the dimensionality and morphology of the superlattice. For instance, 2D layered superlattices often rely on weak interlayer van der Waals or electrostatic forces, whereas 1D and 3D nanoparticle‐based superlattices are governed by stronger chemical interactions, including covalent, ionic, and ligand‐mediated van der Waals forces. Despite their utility, each synthesis method presents notable limitations. Solid‐substrate assembly frequently suffers from limited scalability and non‐uniform domain formation. Liquid‐interface assembly is highly sensitive to environmental fluctuations (e.g., humidity, solvent volatility), resulting in poor reproducibility. Confinement‐guided assembly, although effective in regulating particle orientation and shape, faces challenges in achieving long‐range order and structural uniformity over large areas. Moreover, all three approaches still lack precise control over interparticle spacing and the dynamic behavior at the molecular scale. Overcoming these limitations will require not only an improved synthetic control strategy but also a more comprehensive mechanistic understanding of the spatial and temporal aspects of the self‐assembly processes.

Soft ligands have become an essential design element in the fabrication of superlattice materials, offering precise control over nanoparticle interactions and spatial organization. A wide range of soft ligands, including small molecules, polymers, and DNA, have been employed to direct the self‐assembly of NPs into highly ordered superlattice structures [83]. Among them, molecular ligands such as alkanethiols exhibit strong affinity for metallic nanoparticle surfaces through covalent bonding, which enhances the structural stability of the assembled frameworks during self‐organization [84]. In parallel, steric repulsion between adjacent ligands can effectively counteract the strong core‐to‐core van der Waals attractions, thereby facilitating ordered assembly, particularly in solid‐substrate‐based self‐assembly processes. Under thermodynamics equilibrium, these strong van der Waals interactions often lead to ligand interdigitation, further stabilizing the superlattice architecture [85, 86]. In addition, tuning the ligand structure, especially the chain length and terminal functional groups, provides a practical strategy to regulate interparticle spacing. For example, organic thiols with chain lengths ranging from C_6_ to C_20_ have been systematically used as capping ligands. Longer chains increase the distance between particles, resulting in looser packing and weaker interparticle coupling [51, 87]. However, the detailed mechanisms by which soft ligands influence superlattice formation at the molecular scale remain poorly understood. Factors such as ligand flexibility, solvation effects, dynamic rearrangement, and interaction with the solvent environment introduce significant complexity, making it challenging to predict or fully control the assembly process and its subsequent impact on surface accessibility.

To elucidate the molecular‐level mechanisms behind ligand‐mediated superlattice formation, in‐situ nano‐FTIR has emerged as a powerful tool. This technique enables real‐time monitoring of chemical and structural changes at the nanoscale during the synthetic process, providing direct insight into the dynamics of ligand binding and functionalization on nanoparticle surfaces. Additionally, combined with atomic force microscopy (Figure 7b), which acts as an antenna to generate a strong infrared field near the apex, this technique can achieve high spatial resolution (< 20 nm) with surface and chemical sensitivity. By identifying the vibrational modes of chemical bonds, the composition of various ligands can be precisely characterized, leading to a more comprehensive understanding of superlattice formation. For example, tetradecylphosphonic acid (TDPA), a widely used ligand in superlattice synthesis, exhibits distinct peaks in the P–O stretch region (Figure 7c), serving as an infrared fingerprint for the process. Spectra taken under different bias conditions can reveal bias‐induced intensity changes in the P–O mode (Figure 7d). In the initial state, the presence of the ν_as_(P═O) mode indicates TDPA binding to the Ag surface in a bidentate mode. Changes in the intensity of the ν_as_(P–O) mode reflect bias‐induced structural evolution, offering a detailed look at the molecular‐level interactions in shaping nanoparticle functionalities [88]. These insights not only deepen our understanding of ligand coordination but also provide a foundation for tailoring surface chemistry to optimize nanoparticle assembly. However, while ligands facilitate nanoparticle dispersion and ordering, they may also weaken electronic or thermal coupling between adjacent units [89]. This trade‐off highlights the need to develop new ligand systems or post‐processing strategies to preserve interparticle connectivity without compromising structural integrity.

While experimental methods provide important real‐time observations of nanoparticle assembly, integrating them with computational simulations is essential for capturing detailed atomic configurations and dynamic transformation pathways. In particular, machine learning‐assisted simulations enable efficient exploration of complex energy landscapes and offer predictive capabilities for materials with tunable architectures. This approach is especially well‐suited to superlattice systems, where well‐defined structural units are repeated periodically. By modeling interactions at the atomic level, these simulations help optimize unit design, interface matching, and energy profiles, providing guidance for achieving desirable collective properties. For example, the Stochastic Surface Walking (SSW) global optimization algorithm, combined with a neural network potential (SSW‐NN), has been used to map the phase diagram of Cu_x_O. This method reveals atomic arrangements, interfacial lattice matching, and energy barriers between different facets of Cu_2_O. Such insights are directly applicable to the design of modular units for superlattice construction, enabling programmable control over structure and function [90]. By integrating Nano‐FTIR with such computational modelling, researchers can transition from trial‐and‐error synthesis to a predictive fabrication roadmap. This combination is critical for identifying the molecular‐level interactions that govern long‐range order, thereby providing a direct feedback loop to optimize the reproducibility of complex architectures.

Despite recent progress, microscopic techniques for probing the synthesis and structural evolution of superlattices remain underdeveloped. Given the complexity and tunability of superlattice architectures, this limitation creates significant knowledge gaps in understanding the relationship between superlattice structure and properties. As a result, both the exploration of their full application potential and the advancement toward scalable manufacturing are significantly constrained. In overcoming these barriers, coordinated efforts in both experimental characterization and theoretical modeling are essential for enabling rational design and practical implementation of superlattice‐based systems.

### 4.2 Charge Transfer Observation

Photothermal catalytic activity is intricately linked to charge dynamics within periodic structures, making the understanding of nanoscaled charge separation mechanisms crucial for optimizing full‐spectrum solar energy harvesting and enhancing catalytic performance. Directed charge transfer, a key focus in photocatalysis, not only minimizes electron–hole recombination for enhanced carrier utilization but also improves reaction specificity and product selectivity. As previously discussed, the unique structural features of superlattices introduce a range of unconventional charge behaviors, such as periodic potential modulation, inter‐unit hopping, interfacial state transport, and quantum tunnelling, which collectively enhance their photothermal catalytic potential. In particular, the precise control over inter‐unit spacing and composition allows for the engineering of directional charge transfer pathways, effectively guiding the migration of electrons and holes across the structure. However, these processes typically occur on ultrafast timescales (picoseconds or less) and at the nanoscale, rendering them difficult to resolve using conventional techniques. Moreover, under realistic catalytic conditions, especially those involving thermal excitation induced by photothermal conversion, charge behaviors may deviate significantly from those predicted by idealized static crystal models. Therefore, advanced characterization techniques capable of probing charge dynamics in situ and under operating conditions are essential for elucidating the true mechanisms underlying superlattice‐based photothermal catalysis.

Recent advances in spatially resolved surface photovoltage (SRSPV) methods have provided new tools for studying the charge transfer behaviors. This technique utilizes anodized conductive tips to extract changes in surface photovoltage (SPV), which reflects variations in the light‐induced surface potential, using the Kelvin probe method [91]. An external lock‐in amplifier is employed to enhance sensitivity [92]. According to the Poisson equation and charge center method, the magnitude of the SPV signal corresponds to the density and separation distance of the generated charges, while the sign of the SPV indicates the direction of charge separation. Thus, the SRSPV signal is closely related to nanoscale charge separation processes, enabling direct mapping of the distribution of photogenerated charges on the surface of photocatalyst particles and quantitative determination of the driving force behind photogenerated charges.

**FIGURE 8:**
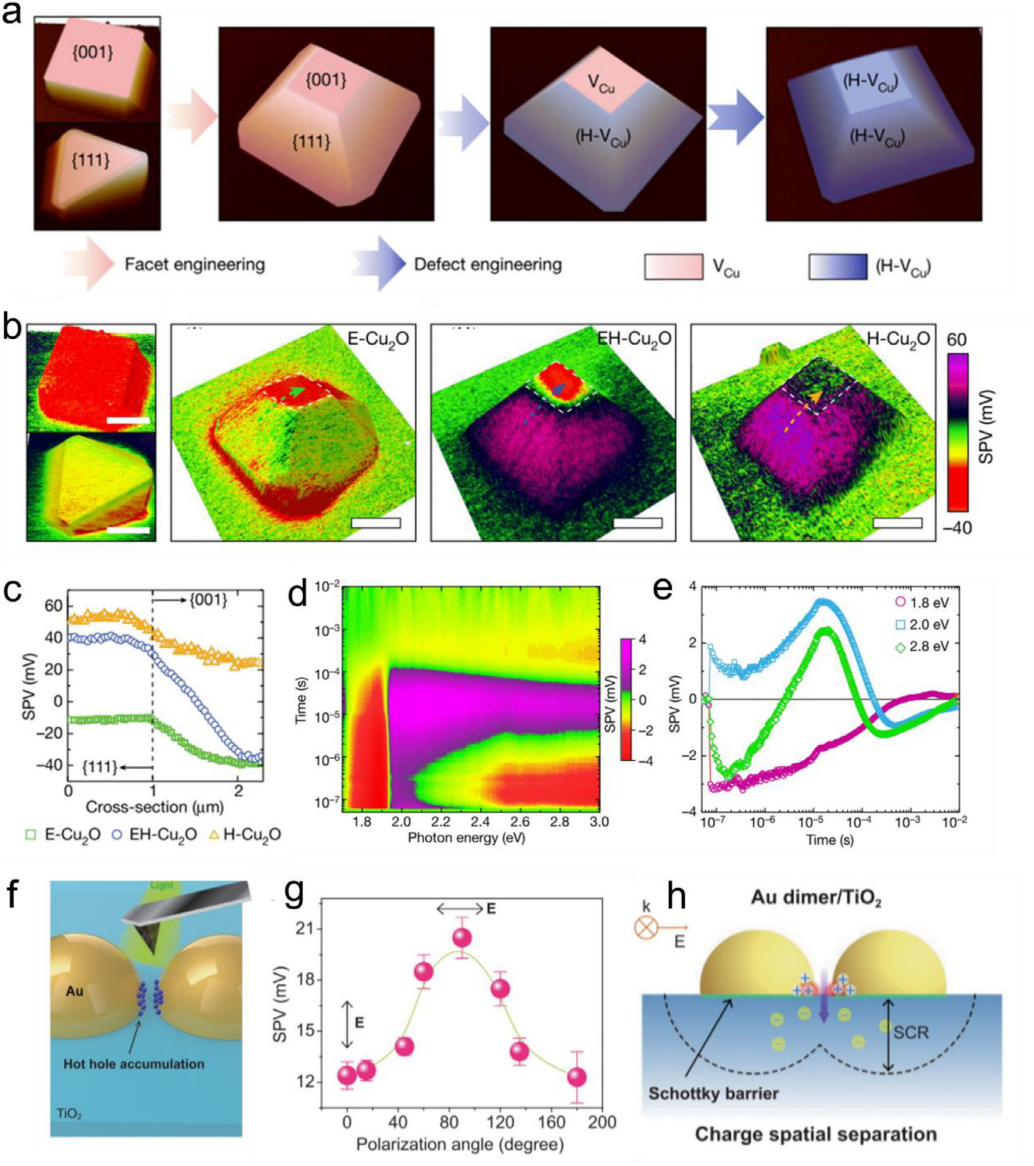
(a) Illustration of the anisotropic engineering of facets and defects [94]. (b) SPVM images of particles [90]. (c) SPV values extracted across dashed lines [94]. (d) Image of the spectral and time‐dependent distributions of SPV signals [94]. (e) SPV transients of particles extracted from photon energies of 1.8, 2.0, and 2.8 eV [94]. Reproduced with permission [90]. Copyright 2022, Nature. (f) Schematic of KPFM measurement of NDs on the substrate [95]. (g) SPV measured at the spot of interface as a function of incident light polarization angle [95]. (h) Schematic illustration of plasmon‐induced charge spatial separation [91]. Reproduced with permission [95]. Copyright 2018, National Science Review.

SRSPV encompasses techniques such as spatially resolved surface photovoltage spectroscopy and SPVM [93]. Building on the relationship between SPV magnitude and sign, spatiotemporal‐resolved SPVM has evolved from simple potential mapping to a powerful diagnostic platform for elucidating charge transfer pathways. For example, introducing defects on the {001} and {111} surfaces of copper oxide (Figure 8a) leads to the accumulation of photogenerated electrons and holes at different surfaces, which can be measured by SPVM (Figure 8b). Besides, increasing the {111}/ {001} ratio leads to a stronger SPV signal, confirming the accelerated charge transfer separation (Figure 8c). Additionally, transient SPV spectroscopy analysis provides insights into the charge transfer process on a nanosecond timescale (Figure 8d, e). At excitation energies above the bandgap (2.04 eV), the SPV signal initially appears negative on the nanosecond scale, shifting to positive over microseconds. The negative SPV signal results from hole capture and electron drift toward the {001} plane, while the positive SPV signal on the {111} crystal face reflects electron capture by defects. These defects are likely located within a depth of 60 nm below the {111} surface, thereby enabling local hole transfer to the {111} plane. Hole and electron capture time spans nanoseconds and tens of microseconds, respectively, consistent with the inverted SPV signal observed during super‐bandgap excitation [94]. Understanding the distinct characteristics of different crystal planes in various materials allows for the optimization of superlattice design to maximize photo‐induced electron and hole separation and their transfer efficiencies. By leveraging these insights, superlattices can be engineered with tailored surface chemistries and structural features to optimize their electronic properties and enhance overall performance in photothermal catalytic processes.

In superlattices, the coupling between repeating units generates a coupling electric field at the junctions, influencing electron migration. Quantifying this coupling electric field is also important to design and construct a giant and directional driving force on hot carriers. KPFM, a high‐resolution scanning probe microscopy technique, excels at measuring surface potential with exceptional spatial precision. Based on the Kelvin probe principle, KPFM detects the contact potential difference between a conductive probe tip and the sample surface, allowing for precise characterization of electronic properties at the nanoscale. KPFM has been employed to investigate coupling electric fields in model systems such as nanoparticle dimers (NDs), where it enables the analysis of hole accumulation within nanogap regions (Figure 8f). By combining with an excitation light source, KPFM images the distribution of surface potential changes, which is directly related to the spatial distribution of separated charges. Under different polarizations, the SPV will be induced by plasmonic excitations (Figure 8g). The observed SPV exhibits a sinusoidal shape in the range of 0° to 180°, with the maximum value at 90°, indicating the polarization direction of the electromagnetic field in relation to the axis between the particles (Figure 8h). This observation demonstrates that the plasmonic coupling effect at the interface provides a strong near‐field within the nanogap region, leading to high‐density accumulation of spatially separated holes at the hotspots [95]. By leveraging KPFM, these phenomena can be quantitatively resolved, providing detailed insight into local electric field distributions and charge dynamics. While NDs serve as simplified model systems for understanding localized plasmonic interactions, KPFM further enables the visualization of how these local near‐fields evolve into collective electronic states across the periodic landscape of a superlattice. In particular, by mapping coupling electric fields at the junctions of repeating units, KPFM reveals long‐range charge‐transfer pathways that are either enhanced or constrained by lattice symmetry.

Despite the progress in spatial and temporal characterizations of charge dynamics within superlattice structures, several critical challenges remain. Most studies focus on simplified or low‐dimensional models, whereas real‐world superlattices often possess complex three‐dimensional or hierarchical architectures with multidirectional coupling effects. Furthermore, current characterization tools still face limitations in capturing ultrafast carrier–phonon interactions and in situ interfacial dynamics. Addressing these challenges will require integrated advances in ultrafast spectroscopy, nanoscale imaging, and multiscale simulations to construct a comprehensive understanding of charge behavior in photothermal catalysis by superlattices.

### 4.3 Localized Temperature Determining

Photothermally generated heat is equally critical to photothermal catalysis, as local thermal energy not only activates reaction pathways by lowering energy barriers, but also modulates charge transport, intermediate stability, and reaction kinetics through coupled thermal–electronic effects. Superlattices, with their periodic architectures, exhibit distinctive thermal behaviors, such as photon confinement, interfacial phonon scattering, and anisotropic thermal conductivity, which collectively promote efficient heat generation and retention. By virtue of their periodic architecture, superlattices allow for precise modulation of inter‐unit spacing and composition, thereby enabling the design of structured thermal transport channels to concentrate heat spatially. These thermal gradients can induce internal electric fields via the Seebeck effect, further influencing charge dynamics and overall catalytic performance. However, the heat distribution in superlattices occurs at a nanoscale and dynamically responds to irradiation, and deviations from uniform thermal fields may lead to localized overheating or underheating, impairing reaction selectivity and efficiency. Moreover, layer thickness ratio, repetition period, and the spatial arrangement of heterogeneous units directly influence thermal coupling behaviors. These effects are not simply linear superpositions but rather emerge from complex spatial interactions. To truly understand the periodic correlation between structure, heat distribution, and catalytic performance, localized thermal imaging and microscale temperature analysis are indispensable, not only for effective thermal management, but also for tuning coupled thermal‐electronic processes and maximizing the catalytic potential of superlattices.

**FIGURE 9:**
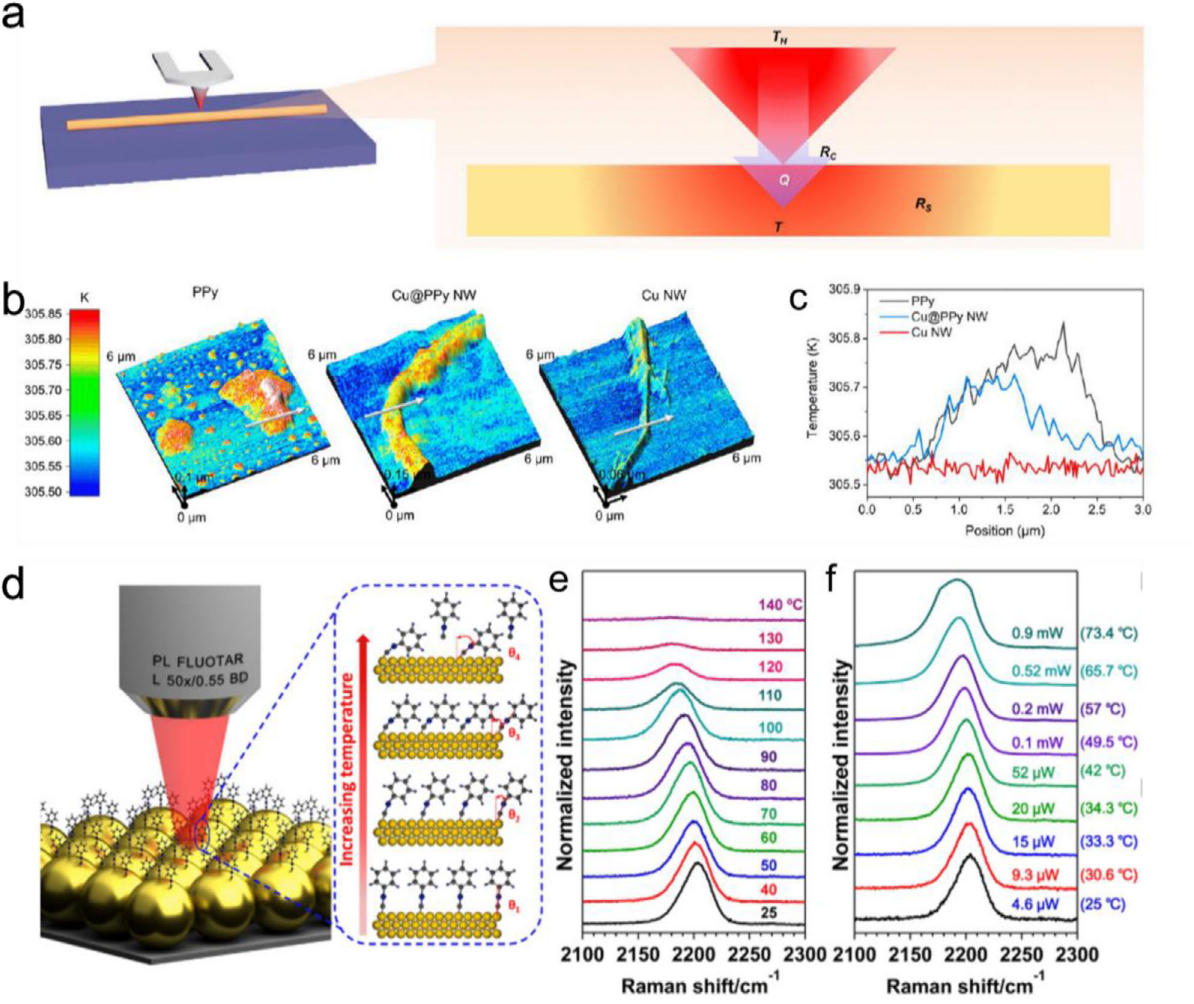
(a) Simplified diagram of SThM via a probe heating mode and thermal resistance across the tip‐sample surface interface [96]. (b) SThM mapping of polypyrrole (PPy), Cu nanowire (NW), and Cu@PPy NW [96]. (c) Section lines in SThM mappings along the white arrows [96]. Reproduced with permission [96]. Copyright 2021, National Science Review. (d) Schematic of temperature‐dependent SERS measurement and temperature‐induced orientation change of PIC molecules [100]. (e) Temperature, and (f) Laser power‐dependent SERS spectra of PIC in the spectral range of the N≡C stretching vibration [100]. Reproduced with permission [100]. Copyright 2021, Journal of the American Chemical Society.

SThM is a powerful technique for high spatial resolution temperature measurements at a micro‐ and nanoscale. It operates by scanning a thermal probe across the sample surface (Figure 9a), where local heat fluxes distributed within the region are detected. This causes changes in the probe's temperature and resistance, and a measurable voltage change and an imbalance in the Wheatstone bridge circuit. SThM can detect temperature variations across different sites (Figure 9b,c), offering valuable insights into optimizing photothermal effects within superlattices [96]. A key advantage of SThM lies in its ability to validate whether engineered superlattice periodicity effectively confines heat at catalytically active sites. By identifying the exact locations of localized hotspots, SThM allows optimization of inter‐unit spacing and layer thickness to maximize thermal accumulation at reaction interfaces, thereby lowering activation barriers for kinetically demanding bond transformations. However, the accuracy of SThM depends heavily on the thermal probe. Although current probes are nanoscale, their size limits spatial resolution, prompting ongoing research into smaller probes for greater precision. Additionally, thermal coupling between the probe and sample can cause cross‐interface interference during measurements. This issue is particularly challenging in superlattices, where strong coupling between the repeating units may distort the results. Overcoming such interference is a key challenge for future studies [97].

Raman microscopy has also been employed for temperature measurements. However, achieving accurate microscale measurements can be difficult due to the small frequency shifts and weak Raman signals in bulk materials. Advanced techniques like surface‐enhanced Raman spectroscopy (SERS) and TERS improve temperature determination by using the intensity or intensity ratio of two Raman peaks, but these methods can be influenced by the coupling state of NPs, which may compromise their accuracy [98, 99]. As such, Hu [100] proposed a local temperature sensing method using phenyl isocyanide (PIC) molecules adsorbed on Au NPs (Figure 9d). The N≡C stretching vibration peak of PIC molecules exhibits strong temperature dependence: as the temperature increases, the angle between the PIC molecule and the Au surface increases, weakening the N≡C bond and causing a redshift in the N≡C stretching frequency. This relationship enables the construction of a temperature‐frequency curve (Figure 9e‐f) for measuring the surface temperature of Au NP substrate with high intensity, achieving 0.232 cm^−1^/°C and a precision of 0.43°C.

The sensitivity of PIC molecules to Au NPs is highly specific, and the PIC probe lacks universality and cannot be easily applied to other materials. However, this concept offers inspiration: each metal may have a corresponding compound to exhibit distinct, temperature‐dependent behaviors, reflecting the complexity of nature. With advances in artificial intelligence and sensor technologies, it is conceivable to create comprehensive databases for surface temperature measurements across various materials [101]. By developing material‐specific sensors, precise correlation curves between temperature and internal vibration frequencies could enable more reliable temperature measurements in a wide range of applications.

In summary, the strategic integration of advanced characterization techniques establishes a comprehensive diagnostic platform for superlattice engineering. Nano‐FTIR, when combined with AI‐assisted modeling, provides molecular‐level insight into ligand coordination and interfacial stability, enabling the rational and reproducible synthesis of high‐quality periodic architectures. Meanwhile, SPVM and KPFM resolve the spatial distribution of surface photovoltage and coupling electric fields, offering critical information on charge dynamics and guiding the optimization of directional carrier transport across repeating units. Furthermore, the synergy between SThM and TERS enables high‐resolution thermal mapping and interfacial temperature profiling, facilitating precise control over heat localization at catalytically active sites. By correlating these microscopic insights with macroscopic catalytic performance, key structural parameters, such as inter‐unit spacing, layer thickness, and facet orientation, can be systematically tuned to maximize energy‐to‐chemical conversion efficiency. Collectively, this integrated, data‐driven approach transforms superlattice design from empirical trial‐and‐error into a rational, performance‐oriented paradigm, thereby accelerating the development of efficient and scalable photothermal catalytic systems.

## 5 Advancing Photothermal Catalysis

Currently, environmental and energy challenges, including carbon utilization [102] (CO_2_‐to‐fuels/chemicals), sustainable nitrogen cycling (NH_3_ synthesis, and nitrate/NO_x_ remediation) [103], green hydrogen production (water splitting) [104], and biomass upgrading [105], demand catalytic systems that operate efficiently, selectively, and robustly under realistic conditions. Superlattice architectures are uniquely positioned to address these demands. Their periodic interfaces, tunable band offsets/dipoles, controllable thermal gradient, and programmable defect fields offer lattice‐level handles to activate inert bonds, stabilize key intermediates, and direct energy flow toward productive reaction pathways. In general, their catalytic performance can be distilled into three central objectives: (i) boosting intrinsic reactivity, (ii) steering selectivity toward desired products, and (iii) upgrading product value through tandem or cascade reactions. In the following sections, we organize emerging applications of photothermal superlattice catalysts along these three axes. This reaction‐centric perspective shifts the focus from materials descriptions to functional catalytic outcomes, revealing how lattice‐level design principles can be systematically aligned with diverse chemical demands.

### 5.1 Boosted Reactivity

**FIGURE 10:**
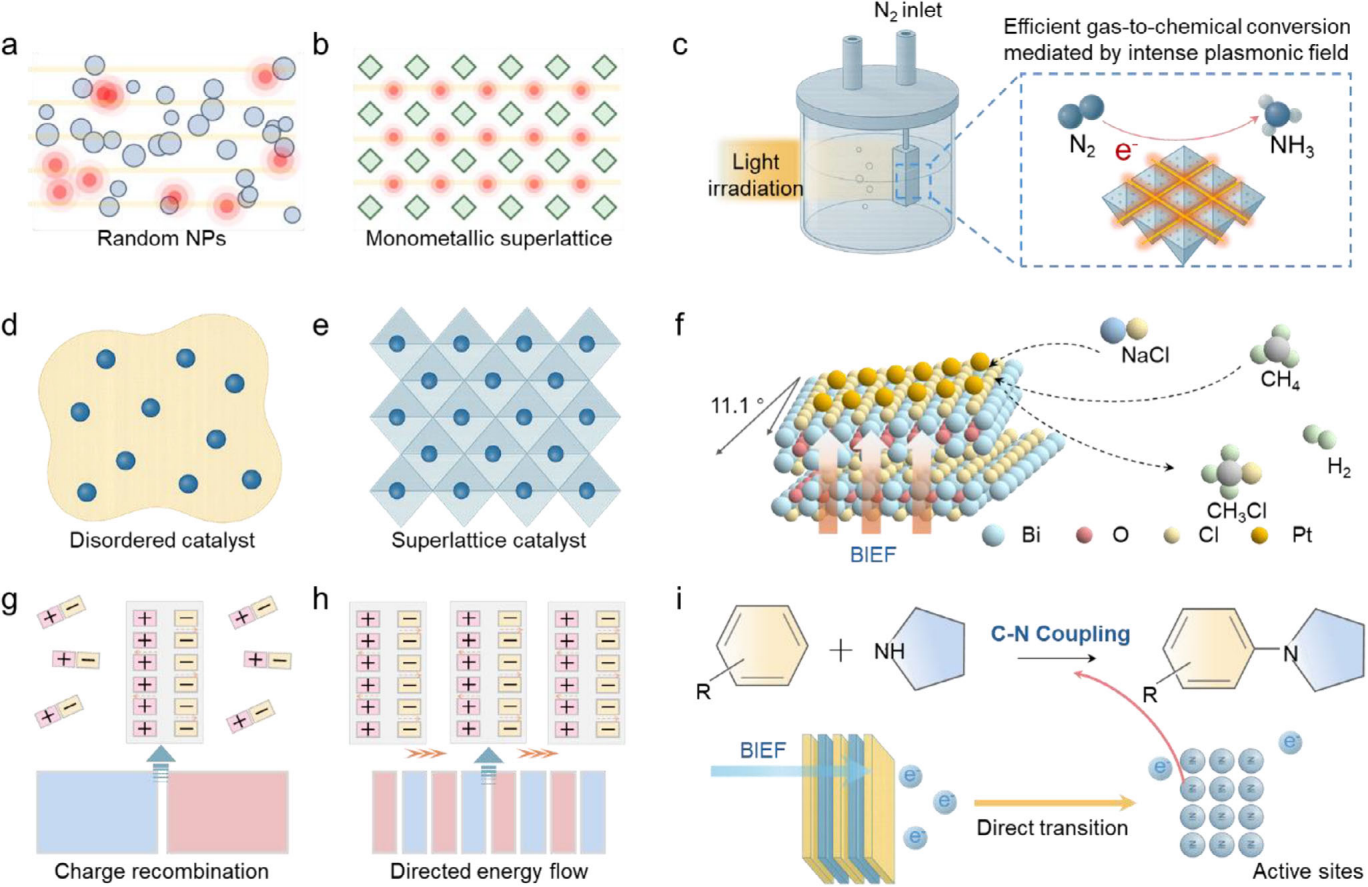
(a) Disordered nanoparticles with sparse stochastic hot spots. (b) Ordered metallic superlattice with collective hot spots. (c) N2 activation under an Ag superlattice. (d) Disordered catalyst with heterogeneous active sites. (e) Superlattice catalyst with equivalent periodic active sites. (f) Layered superlattice achieving ultrahigh CH3Cl selectivity. (g) Single heterointerface polarization and rectified carrier flow. (h) Stacked superlattice with directed energy flux. (i) Heterostructured superlattice with a directional carrier flow for C–N coupling.

The foremost objective of catalysis is to drive reactants across activation barriers toward sustained turnover. Yet many energy‐intensive transformations, including N≡N cleavage, CO_2_ reduction, and oxygen evolution reactions, are governed by strong bonds, multi‐electron transfer, and complex transition states, leading to sluggish kinetics and long induction periods under mild conditions. Conventional bulk heating further exacerbates these limitations by broadening side‐reaction windows and dissipating energy diffusely rather than concentrating at catalytically relevant motifs. These challenges highlight the need for a catalyst architecture that can localize energy precisely on active sites where chemistry occurs and retain it long enough to impact elementary steps. Photothermal catalysis directly addresses this requirement by confining optical dissipation and heat generation to the nanoscale catalytic motifs, thereby tightly coupling energy input to reaction coordinates. Among photothermal strategies, plasmonic resonances have emerged as a cornerstone. Metallic nanostructures exhibit intense optical responses that efficiently convert incident photons into hot carriers, electromagnetic near fields, and localized hotspots, amplifying visible‐NIR absorption and establishing stable, tunable local temperature windows to lower activation barriers [106]. However, randomly dispersed NPs seldom realize collective enhancement, as they lack deterministic interparticle coupling, so the achievable field intensity, hot‐carrier flux, and local temperature are limited and variable from site to site (Figure 10a). By contrast, metallic superlattices leverage periodic coupling and cooperative near‐field interactions to superimpose fields across repeat units, yielding high‐density, high‐intensity hot spot grids and stronger carrier injection at the catalytic motifs. Thus, superlattice builds a structurally encoded route to concentrating optical, thermal, and electronic driving forces, enabling selective and scalable photothermal catalysis [107] (Figure 10b).

Arranging metallic nano‐units into ordered superlattices converts a sum of single‐particle responses into a programmable collective resonance. This collective coupling can be precisely engineered through multiple channels: (1) gap‐mode coupling, where reduced interparticle spacing amplifies local electric and thermal fields [108]; (2) lattice resonances dictated by array symmetry, synchronizing dipoles over long length scales [109]; (3) orientation‐selective coupling, such as face‐face, edge‐edge, and especially tip‐to‐tip, funnels energy into apex‐localized hot spots, maximizing hot spot density [110]. Guided by this structural logic, Jeong et al. constructed tip‐to‐tip‐oriented 3D octahedral plasmonic superlattices, in which geometric apices host collective hot spots, yielding strong near‐field concentrations. Electromagnetic simulations show that the tip‐to‐tip lattice symmetrizes the surface charge into dipole‐dominant modes and confines the field at the apices, boosting the apex near‐field to |*E*|/|*E*_0_ ≈ 261, compared to 124 in close‐packed nanogaps [111].

Then, Boong et al. demonstrated that plasmonic superlattices can drive N_2_‐to‐NH_3_ conversion under ambient conditions. The catalyst comprises ordered Ag metal superlattices for concentrating light through gap‐mode coupling and periodicity‐induced lattice resonances, superimposing electromagnetic near fields and nanoscale heating across repeat units via edge–edge coupling (Figure 10c). Under mild conditions, this superlattice delivers an NH_3_ formation rate of 59.2 µmol·h^−1^·g^−1^ from N_2_, approximately four times higher than that of a disordered Ag assembly. Electron/thermal scavenger tests disentangle the synergistic roles of hot‐electron injection and localized photothermal heating in weakening the N≡N bond. Owing to orientation and uniform nanogaps, the superlattice preserves intense yet spatially smoothed temperature profiles, enabling stable and reproducible ammonia production [52].

### 5.2 Selectivity

Another central bottleneck in translating photothermal catalysis into industrially viable processes is the difficulty of achieving high product selectivity. Broad product distributions impose cascading penalties, including diluted target yields, increased separation and purification costs, and accelerated catalyst deactivation. Fundamentally, a selectivity loss in photothermal systems arises from three intertwined factors [112, 113, 114]. First, macroscopic or non‐localized heating indiscriminately supplies thermal energy, unintentionally opening parallel side‐reaction pathways. Second, structural and site heterogeneity in randomly assembled NPs decouple electromagnetic hot spots and reactive fields from true catalytic motifs at the micro‐ and nanoscale, directing different regions of the catalyst along divergent reaction pathways (Figure 10d). Third, spatiotemporal mismatch of charge carriers occurs because hot electrons and holes are often generated far away from target sites, injected with inappropriate energies, or not coinciding with the lifetime windows of key elementary steps. Consequently, improving selectivity is not simply a matter of adjusting reaction temperature, while it requires structured energy delivery, supplying light, heat, and charge carriers to the right location, with the matched intensity and at the correct time.

Superlattice architecture intrinsically encodes such constraints into its periodic frameworks, offering an efficient route to selectivity control. In plasmonic metal superlattices, uniform nanoscale gaps not only amplify electromagnetic near fields but also establish intense yet spatially smoothed microscopic thermal windows. This deterministic geometry focuses energy on a programmable manner onto catalytically relevant sites, stabilizing the local reaction environment (Figure 10e). The precise control over the gap size and orientation enhances the spatial co‐localization of adsorbates and reactive charge carriers, increasing the probability of intended elementary steps while depriving competing pathways of excess electrons and heat. Similarly, in layered semiconductor superlattices, interlayer coupling and periodic potentials partition electrons and holes into distinct spatial channels, routing them toward designated chemical motifs and suppressing parallel reactions [115]. These periodic potentials confine reactions within a narrow and reproducible electrochemical window, mitigating over‐reduction, over‐oxidation, and thermally driven chain reactions.

A compelling demonstration of this spatiotemporal window engineering is presented in twisted BiOCl Moiré superlattices [116]. Introducing a twist angle of ∼11° generates a Moiré potential and periodic strain field that concentrates holes at Bi‐Cl motifs, promoting lattice‐chlorine activation to ·Cl radicals for selective C‐H/Cl coupling. Concurrently, enhanced interlayer coupling directs electrons toward isolated Pt single‐atom sites, enabling rapid H_2_ evolution and preventing electron accumulation. This functional partitioning of charge carriers, combined with a periodically confined electrochemical window, effectively suppresses over‐chlorination and other competing pathways. Under visible‐light irradiation in the presence of NaCl, the catalyst system achieves ∼96% selectivity toward CH_3_Cl with a formation rate of 53 µmol·g^−1^·h^−1^, demonstrating that superlattice‐encoded carrier routing and window control can directly translate into high and durable selectivity (Figure 10f).

### 5.3 Upgrading Product Value

Once high efficiency and selectivity are established, upgrading product value becomes the critical step for translating laboratory advances into a tangible process with economic benefits [117]. Advancing along platform molecules such as CO, CH_3_Cl, and NH_3_ through further coupling, functionalization, or directed rearrangement can markedly increase value density per unit carbon or nitrogen and widen profit margins per unit product, while reducing the separation and disposal burden associated with low‐value byproducts [118, 119]. Converting these products into energy carriers or storage‐friendly forms, such as liquid fuels, polymerizable monomers, or fine‐chemical precursors, can improve storage and transport safety, lower the carbon footprint and other life‐cycle metrics, and enhance resilience to market fluctuations [120]. Superlattices are well‐suited as valorization platforms because they translate structural order into pathway control, coordinating step‐specific activation and product routing within a single material to complete tandem upgrades. In practice, tuning lattice resonances and gap geometry makes each elementary step match its own operating spectrum and local thermal window, delivering stepwise excitation and temporal alignment [121]. Within this structural framework, spatial routing can shuttle the product of the first step in situ to the second reaction site, biasing pathways and completing tandem or cascade transformations. At an array scale, uniform microscopic thermal windows and equivalent site distributions help preserve reproducible selectivity and yield upon scale‐up, providing a reliable foundation in materials and engineering.

A representative example is the polarization‐ordered layered superlattice Bi_4_TaO_8_Cl‐Bi_2_YO_4_Cl, which encodes the sequence “directional energy → tiered tasking → tandem bond construction” into its A|B|A|B… periodicity [122]. Work‐function offsets, band‐edge alignment, and spontaneous layer polarization collectively generate a stable field along the stacking direction. This vectorial field drives unidirectional carrier transport, reduces exciton binding energy, suppresses bulk recombination, and significantly extends carrier lifetimes (Figure 10g). As a result, vertical functional partitioning emerges: the upper sublayer harvests light and activates substrates while continuously supplying electrons, whereas the lower sublayer couples spatially to a Ni catalytic cycle to complete C─N bond formation via oxidative addition, ligand reorganization, and reductive elimination under a directed electron flux and a mild thermal window. Under visible‐light irradiation, this polarization‐programmed tandem system achieves 99% conversion and 99% selectivity in pyrrolidine‐4‐bromobenzonitrile coupling (Figure 10i), demonstrating that “interfacial polarization rectification plus tiered tasking” can orchestrate multistep chemistry into a controlled tandem process. This paradigm establishes a generalizable blueprint for amination, etherification, and cross‐coupling reactions in superlattice systems.

Further illustrating functional partitioning in application‐oriented architectures, an integration of a superlattice interface with an S‐scheme heterojunction highlights how distinct reaction zones can be encoded within a single material [123]. The superlattice interface introduces periodic potentials, BIEFs, and interfacial diploes, while the S‐scheme band alignment enforces selective recombination. Together, these features are theoretically expected to promote ultrafast charge separation and directional carrier transfer. At the interfacial “weak side”, low‐energy electrons and holes preferentially recombine, while the “strong side” preserves high‐energy conduction‐band electrons and high‐energy valence‐band holes. Consequently, the preserved electrons efficiently drive H_2_ evolution, while the holes are rapidly consumed by sacrificial S^2−^/SO_3_^2−^ species, creating spatially separated redox sites that suppress back reactions and parasitic pathways and thereby enhance cocatalyst‐free H_2_ production efficiency. This creates a clear spatial division between reduction and oxidation sites, reduces useless thermalization, and suppresses side reactions. Although it is not a strict tandem reaction, this approach of interface engineering and band programming provides a clear route to functional zoning within one structure and offers a directly transferable design and characterization paradigm for advancing from partitioned zones to on‐site tandem processes.

Tacking together, superlattices align naturally with the three pillars required for industrial deployment: throughput, selectivity, and downstream valorization. By structurally encoding the manners of photons, heat, and charge carriers to be generated, confined, and delivered, superlattices create dense hot‐spot networks for high reaction rates, restrict chemistry to narrow and reproducible operating windows for selectivity, and enable spatial handoff for tandem upgrading. Although large‐scale demonstrations remain limited, the strong correspondence between reaction requirements and the tunable structural levers of superlattices points to a highly promising trajectory for advancing photothermal catalysis toward practical implementation.

## 6 Conclusion, Challenge, and Perspective

In this article, we provide an in‐depth review of diverse superlattices as a unifying platform to advance photothermal catalysis. We highlight their unique photoelectrochemical properties, strengthened photothermal behavior, and improved engineered reaction microenvironments for translation into boosted reactivity, improved selectivity, and upgraded product value. We also summarize advanced characterization techniques tailored to photothermal catalysis in superlattices, offering tools for accurate mechanism studies and rational structure‐performance correlation. Together, these advances position superlattices not merely as model systems, but as sophisticated design frameworks capable of elevating photothermal catalysis from early‐stage laboratory demonstrations to efficient, value‐oriented solar chemical transformations. However, moving toward practical implementation requires addressing critical hurdles such as synthetic complexity, long‐term structural stability under photothermal conditions, and the scalability of ordered architectures. Recognizing these challenges not only provides a realistic outlook but also highlights the strategic opportunities for integrating machine learning and advanced manufacturing to overcome these bottlenecks.

Superlattices enable the rational design of their morphological features, allowing for precise control and optimization of their properties. By carefully designing the morphology, size, and structure of crystals, one can tailor the optical, electrical, magnetic, and other properties of the materials to meet the specific requirements for various applications. Traditional experimental methods often demand considerable time and resources to determine the optimal catalyst structure and its stability under extreme conditions. In overcoming these limitations, machine learning (ML) offers a data‐driven alternative capable of accelerating the discovery and optimization of superlattice‐based catalysts. For instance, machine learning interatomic potentials (MLIPs) employ advanced non‐linear regression algorithms to predict energy and force distributions from atomic configurations [124]. When integrated with large‐scale Monte Carlo simulations, MLIPs have been used to reveal size‐dependent surface structure and compositional changes in CuAu NPs, illustrating the complexity of active site ensembles at a microscale [125]. By leveraging ML‐enabled modeling, researchers can efficiently explore design strategies to enhance the performance and durability of superlattices, particularly under high‐temperature or harsh reaction environments. As such, the integration of ML with materials engineering represents a promising direction for next‐generation catalyst design.

During material synthesis, precise control over parameters such as ligand selection, solvent composition, temperature, and reaction time is essential to ensure the desired crystallographic and morphological features. Achieving such fine‐tuned synthesis often requires advanced techniques and instrumentation, posing challenges for scalability and reproducibility. Given these challenges, additive manufacturing techniques, particularly 3D printing, have emerged as a promising strategy for large‐scale catalyst fabrication. Unlike conventional methods, 3D printing offers high flexibility and customizability, enabling the construction of complex architectures with nanoscale precision [126]. This capability is particularly well‐suited to the structural demands of superlattices, allowing for controlled deposition of building blocks with defined spatial arrangements [127, 128]. For instance, direct‐write 3D printing has been used to fabricate Fe_3_O_4_ NPs with uniform 15 nm dimensions [129]. Furthermore, 3D‐printed superlattice films such as Ge_2_Sb_2_Te_5_/Mg_35_Sb_65_ have exhibited excellent mechanical adaptability, retaining their functionality even after bending or extrusion [130]. These examples underscore that 3D printing can bridge the gap between nanoscale design precision and scalable production, unlocking new possibilities for superlattice engineering. However, despite its flexibility, 3D printing still faces significant challenges in achieving the high throughput and cost‐efficiency required for industrial‐scale massive production. Furthermore, maintaining structural consistency over large areas remains technically demanding for the current additive approach.

In addition to 3D printing, several advanced micro/nanofabrication techniques have demonstrated great promise in the construction of superlattice structures. Laser etching, known for its high spatial precision and efficiency, enables precise tuning of geometric features such as void size, shape, and arrangement, thereby offering a reliable pathway to optimize structural and functional performance [131, 132]. Focused ion beam milling, particularly using suspended graphene membranes, has achieved interlayer spacings as small as 16 nm, while simultaneously enabling control over lattice symmetry and periodic strength and paving the way for the fabrication of ultra‐short superlattices [133]. Additionally, displacement talbot lithography (DTL), which employs phase interference patterns generated via optical displacement, offers scalable patterning capabilities. By structuring photoresist layers into periodic templates, DTL demonstrates strong potential for large‐area fabrication of superlattice frameworks [134, 135].

Superlattices hold broad promise for solar‐driven catalysis owing to their architected interfaces, directional energy transport, and tunable active sites. Yet current demonstrations remain narrow in scope and do not fully exploit their capabilities beyond a few model reactions. Looking ahead, systematically deploying diverse superlattice designs across urea synthesis, nitrate/nitrite reduction, CO_2_ conversion, selective oxidations, and tandem cross‐couplings will be critical to translating this platform toward green‐energy and environmental applications. Progress hinges on deeper mechanistic insight, resolving how band offsets, interfacial dipoles, plasmonic/photothermal heating, and thermoelectric effects are coupled to govern charge/heat transport and intermediate stabilization. Priorities include establishing quantitative structure–performance relationships, developing operando spectroscopies and multiscale theory, and benchmarking stability and scalability under realistic conditions. With coordinated experimental and computational efforts, the working principles of superlattices can be fully elucidated, enabling their complete potential to be realized across a wide range of catalytic reactions.

## Conflicts of Interest

The authors declare no conflicts of interest.

## Data Availability

Data sharing not applicable to this article as no datasets were generated or analysed during the current study.
